# Integrative analysis of single‐cell multi‐omics reveals heterogeneity in piriform cortex principal neurons and their selective vulnerability in Alzheimer's disease

**DOI:** 10.1002/ctm2.70781

**Published:** 2026-09-11

**Authors:** Jiayue Meng, Miaomiao Zhang, Yujie Chen, Mengyao Han, Nianhao Cheng, Ruifang Cao, Qinwen He, Yaya Zhao, Liyun Yuan, Guoqing Zhang

**Affiliations:** ^1^ Bio‐Med Big Data Center Shanghai Institute of Nutrition and Health University of the Chinese Academy of Sciences, Chinese Academy of Sciences Shanghai China; ^2^ State Key Laboratory of Immune Response and Immunotherapy Shanghai Institute of Materia Medica University of Chinese Academy of Sciences, Chinese Academy of Sciences Shanghai China; ^3^ State Key Laboratory of Immune Response and Immunotherapy Shanghai Institute of Materia Medica Chinese Academy of Sciences Shanghai China

**Keywords:** Alzheimer's disease, multi‐omics, piriform cortex, principal neurons, selective vulnerability, single‐cell RNA sequencing

## Abstract

**Background:**

The piriform cortex (PIR) is a key region of the olfactory system and is increasingly implicated in Alzheimer's disease (AD). However, the cellular heterogeneity of principal neurons (PN) in this region and their selective vulnerability in AD remain poorly understood.

**Methods:**

Here, we integrated single‐cell multi‐omics datasets to systematically characterize PIR PN heterogeneity. We identified four transcriptionally and epigenetically distinct PN subtypes with different spatial distributions across the anterior and posterior PIR.

**Results:**

In AD mouse models, the abundance of the Reln‐positive superficial‐layer (Reln+ SL) subtype was spatially associated with amyloid‐β pathology, and its reduced proportion was confirmed by multiplex immunofluorescence. Cross‐region comparison revealed that Reln+ SL neurons shared a *Gpc5*‐high/*Sort1*‐low molecular signature with vulnerable layer II excitatory neurons in the entorhinal cortex (EC). In human AD, this vulnerability‐associated signature showed progressive enrichment in EC layer II neurons across Braak stages I–III. Furthermore, A *Sort1*‐containing co‐expression module captured regional differences between lateral and medial EC layer II populations and was enriched for proteostasis‐related pathways.

**Conclusions:**

These findings reveal shared molecular features associated with selective vulnerability across PIR and EC layer II neurons and identify a proteostasis‐related program linked to regional differences in neuronal vulnerability during AD.

**Key points:**

- Single‐cell multi‐omics reveals molecular heterogeneity among piriform cortex principal neurons.
- Reln+ superficial‐layer neurons show selective vulnerability in AD.
- Cross‐region comparison identifies a shared *Gpc5*‐high/*Sort1*‐low signature in vulnerable layer II neurons of EC and PIR.

## Graphical abstract

Integrative single‐cell multi‐omics analysis reveals molecular heterogeneity among piriform cortex principal neurons and identifies Reln+ superficial‐layer neurons as a selectively vulnerable population in Alzheimer's disease. Cross‐region comparison with entorhinal cortex layer II neurons reveals a shared *Gpc5*‐high/*Sort1‐*low vulnerability‐associated signature, and a Sort1‐containing co‐expression module further reveals proteostasis‐related pathways associated with selective vulnerability of layer II neurons in EC subregions.

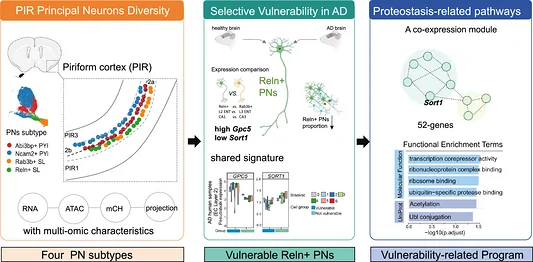

## 1 INTRODUCTION

Alzheimer's disease (AD) is characterized by the selective vulnerability of specific neuronal populations, with different populations exhibiting a gradient of susceptibility to the accumulation of pathological protein aggregates and neuronal degeneration.^1^, ^2^, ^3^ EC is one of the first cortical regions to show atrophy and neuronal loss in AD, contributing to memory impairments.^4^, ^5^ Lateral EC (LEC), which is primarily involved in olfactory information processing,^6^ shows earlier atrophy than medial EC (MEC) in AD.^7^ Neurons within EC layer II, particularly the Reelin‐positive glutamatergic cells,^8^ are highly vulnerable to AD pathology and die early in the course of AD.^1^ Recent single‐cell studies have identified additional populations of vulnerable excitatory neurons that are depleted in specific EC layers in AD,^9^, ^10^ suggesting that selective vulnerability may be associated with neuronal population heterogeneity. Nevertheless, whether these populations exhibit distinct molecular signatures associated with vulnerability remains unclear, particularly regarding the differences between vulnerable neurons in EC layer II and those in other layers. Addressing these gaps is essential for understanding the selective neuronal degeneration that occurs in the early stages of Alzheimer's disease.

Recent studies provide evidence that the Reelin signalling pathway contributes to the selective vulnerability of layer II EC neurons.^9^ Reelin is mostly expressed in GABAergic interneurons in the neocortex.^11^ However, its expression by excitatory neurons is remarkably specific, notably in layer II of the EC and layer II of the PIR, representing a conserved feature in both mice and humans.^12^, ^13^, ^14^, ^15^ The PIR and EC are anatomically and functionally related regions involved in olfactory processing.^16^, ^17^ In the mouse brain, the PIR and EC are directly connected.^18^ In humans, although the PIR and EC are connected via the cortical amygdala,^19^ the superficial LEC receives input from the PIR.^20^ Neurons in EC layers II and III integrate this olfactory input with contextual information before further relaying it to the hippocampal regions.^21^, ^22^ Moreover, olfactory deficits are among the earliest clinical manifestations of AD. Early pathological accumulation has been detected in medial temporal olfactory regions, including the PIR, which also exhibit functional disruption during disease progression.^23^, ^24^, ^25^, ^26^ Given the anatomical and functional relevance of the EC and the PIR, along with their disruption in AD, we raise the possibility that Reelin‐positive neurons in the PIR, like those in layer II of the LEC, may be vulnerable in the early stages of AD. This shared vulnerability may reflect conserved molecular features that distinguish these two populations from relatively resistant neuronal populations within the olfactory cortical circuit. Here, we test this hypothesis using integrative single‐cell multi‐omics analyses to identify shared molecular features that distinguish Reln+ layer II glutamatergic neurons from relatively resistant neuronal populations across the PIR and EC.

Previous studies have primarily identified calretinin‐ or somatostatin‐expressing PIR interneurons that are vulnerable to amyloid‐beta (Aβ) accumulation.^27^ However, whether excitatory neurons exhibit subtype‐specific vulnerability in AD remains largely unexplored. Recent advances in single‐cell technologies^28^, ^29^, ^30^, ^31^, ^32^ and the rapid accumulation of large‐scale multi‐omics data^9^, ^33^, ^34^, ^35^, ^36^, ^37^, ^38^, ^39^, ^40^, ^41^, ^42^, ^43^, ^44^, ^45^ now provide new opportunities to explore this issue in greater depth. Based on these data, we performed an integrative single‐cell multi‐omics analysis of PNs in the mouse PIR and identified four transcriptionally and epigenetically distinct PN subtypes. Spatial co‐localization analysis in AD mouse brain tissue revealed that the Reln‐positive subtype was significantly co‐localized with Aβ aggregates. Multiplex immunofluorescence analysis showed a reduced proportion of this subtype in AD mouse brains. By comparing vulnerable Reln‐positive PNs in the PIR with neurons in layer II of the EC, we identified shared molecular features associated with high *Gpc5* expression and low *Sort1* expression. Furthermore, within EC layer II neurons, we identified a Sort1‐containing co‐expression program that distinguished LEC from MEC populations and was enriched for proteostasis‐related processes. Together, these results advance our understanding of the molecular characteristics of selective neuronal vulnerability in AD.

## 2 RESULTS

### 2.1 Molecular heterogeneity of principal neurons in the piriform cortex

#### 2.1.1 Four transcriptionally distinct PN subtypes in the mouse PIR

**FIGURE 1:**
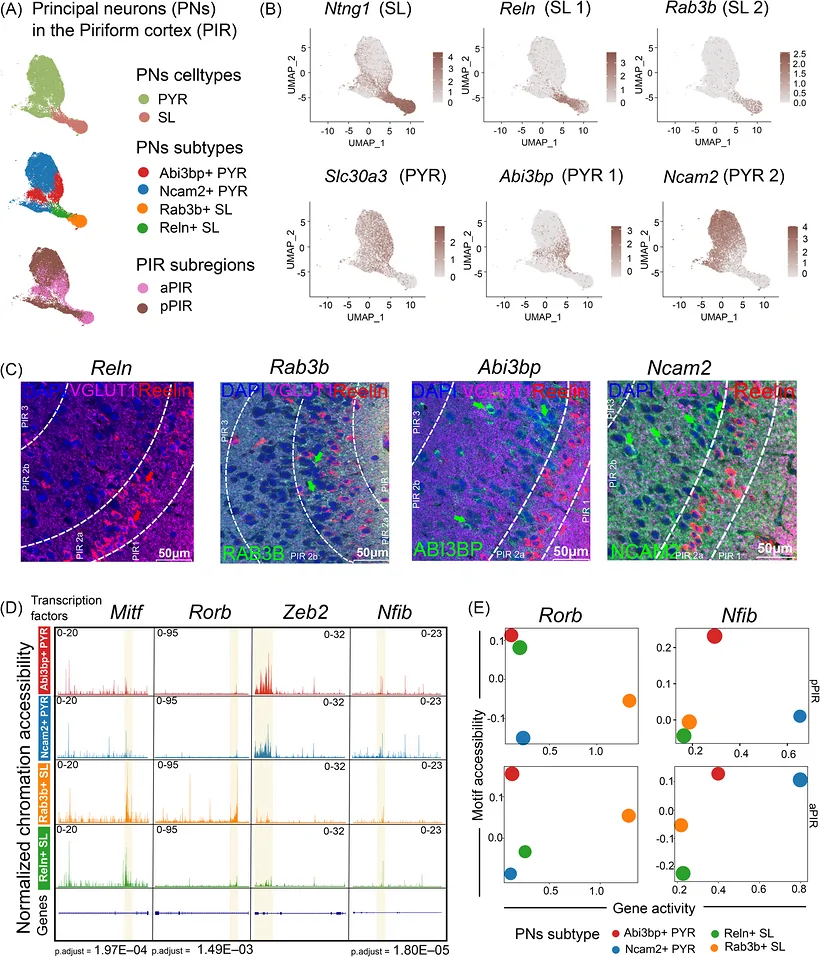
Four PN subtypes and their epigenetic signatures in the PIR. (A) UMAP visualization of PNs, coloured by cell type, subtype and subregion. Data were obtained from a published snRNA‐seq dataset,^33^ comprising 26 975 PIR PNs. SL: Superficial‐Layer; PYR: Pyramidal; aPIR: anterior piriform cortex; pPIR: posterior piriform cortex. (B) Expression of subtype‐specific marker genes defining SL and PYR subtypes. (C) Immunohistochemistry of PN subtype marker expression in the wild‐type mouse PIR. Representative fields of view illustrate co‐staining of the glutamatergic neuronal marker VGLUT1 (magenta) with putative PN markers Reelin (red), RAB3B (green), ABI3BP (green) and NCAM2 (green). Nuclei are counterstained with DAPI (blue). Scale bar, 50 µm (applies to all fields). (D) Genome browser showing chromatin accessibility surrounding selected transcription factors across PN subtypes. Data were obtained from a published snATAC‐seq dataset,^34^ comprising 48 615 PIR PN nuclei. Differentially accessible peaks were identified using the Wilcoxon rank‐sum test, with *p*‐values adjusted using the false discovery rate method. Comparisons were performed as follows: pooled SL (Rab3b+ and Reln+) vs. pooled PYR (Ncam2+ and Abi3bp+) for *Mitf*; Rab3b+SL vs. the other three subtypes combined for *Rorb*; and Ncam2+ PYR vs. the remaining three PN subtypes for *Nfib*. Significance was defined as adjusted *p* value < 0.05 and average log2 fold change > 0.25. (E) Scatterplots comparing transcription factor motif accessibility with corresponding gene activity across PN subtypes in anterior and posterior PIR subregions.

Principal neurons (PNs), defined by high expression of the glutamatergic marker *Slc17a7*, represent the major excitatory populations in PIR.^46^ We identified four transcriptionally distinct PN subtypes by re‐analysing a published single‐nucleus RNA‐seq dataset^33^ comprising 26 975 nuclei from the normal mouse PIR (Figure 1A). Among these subtypes, the superficial‐layer (SL) populations were marked by *Ntng1* expression^46^ and comprised Reln+ neurons enriched in the anterior PIR (aPIR) and Rab3b+ neurons enriched in the posterior PIR (pPIR). In contrast, the pyramidal (PYR) populations were characterized by *Abi3bp* or *Ncam2* expression, and showed high expression of *Slc30a3* (Figure 1B, Figure S1B). RNA in situ hybridization data from Allen Brain Atlas showed that *Rab3b* and *Reln* expression was enriched in superficial layers, whereas *Ncam2* expression was restricted to deeper layers (Figure S1B). Multicolour immunofluorescence further demonstrated the distinct spatial distribution of these subtype markers. Reln+ (SL1) and Rab3b+ (SL2) neurons occupied distinct but complementary positions within the superficial layer (PIR 2a), whereas Abi3bp+ (PYR1) and Ncam2+ (PYR2) neurons were preferentially localized to deeper pyramidal layers (PIR 2b) (Figure 1C, Figure S1A). These findings support the correspondence between transcriptomically defined PN subtypes and their anatomical organization.

We next integrated our snRNA‐seq dataset^33^ with a whole‐mouse‐brain reference atlas^47^ using canonical correlation analysis (CCA) to map our PN subtypes onto established neuronal taxonomies (Figure S5J). Joint embedding and clustering analysis demonstrated correspondence between the four transcriptomic PN subtypes and previously annotated PIR‐lateral entorhinal area (ENTl) populations (Figure S5K). Ncam2+ PYR neurons mainly co‐clustered with PIR‐ENTl 1, 3 and 6, whereas Abi3bp+ PYR neurons clustered with PIR‐ENTl 2 and 7. The superficial‐layer Reln+ SL and *Rab3b*+ SL populations both showed co‐clustering with PIR‐ENTl 4. Thus, our classification is consistent with established PIR PN taxonomy.

#### 2.1.2 Epigenomic signatures associated with PN subtypes

A comprehensive characterization of PN subtype diversity requires complementary transcriptional and epigenetic analyses.^48^ We first examined whether snRNA‐seq‐defined PN subtype markers exhibited distinct epigenetic patterns by leveraging two publicly available single‐cell epigenetic atlases of the mouse PIR (scATAC‐seq^34^ and snmC‐seq2^47^). *Reln*, *Rab3b*, *Abi3bp* and *Ncam2* displayed distinct chromatin accessibility and non‐CG methylation (mCH) levels among unsupervised epigenomic clusters (Figure S1C,D), consistent with their subtype‐specific transcriptional expression patterns.

To identify subtype‐specific transcription factors (TFs), we transferred snRNA‐seq‐defined PN subtype annotations onto the scATAC‐seq dataset^34^ using CCA‐based integration. Differential accessibility analysis of the mapped PN populations identified *Rorb* and *Nfib* as subtype‐associated TFs in Rab3b+ SL and Ncam2+ PYR neurons, respectively (Figure 1D). Both TFs showed subtype‐enriched chromatin accessibility compared with the other three PN subtypes (*Rorb*: FDR = 1.49 × 10^−3^; *Nfib*: FDR = 1.80 × 10^−5^), and their RNA expression was similarly enriched in the corresponding subtypes (Figure S1E). Motif enrichment and gene activity analysis further supported their subtype association, with *Rorb* enrichment in Rab3b+ SL neurons and *Nfib* enrichment in Ncam2+ PYR neurons (Figure 1E). Both *Rorb* and *Nfib* are developmental transcription factors implicated in neuronal identity and cortical development.^49^, ^50^

#### 2.1.3 Spatial differences in PN subtype distribution

**FIGURE 2:**
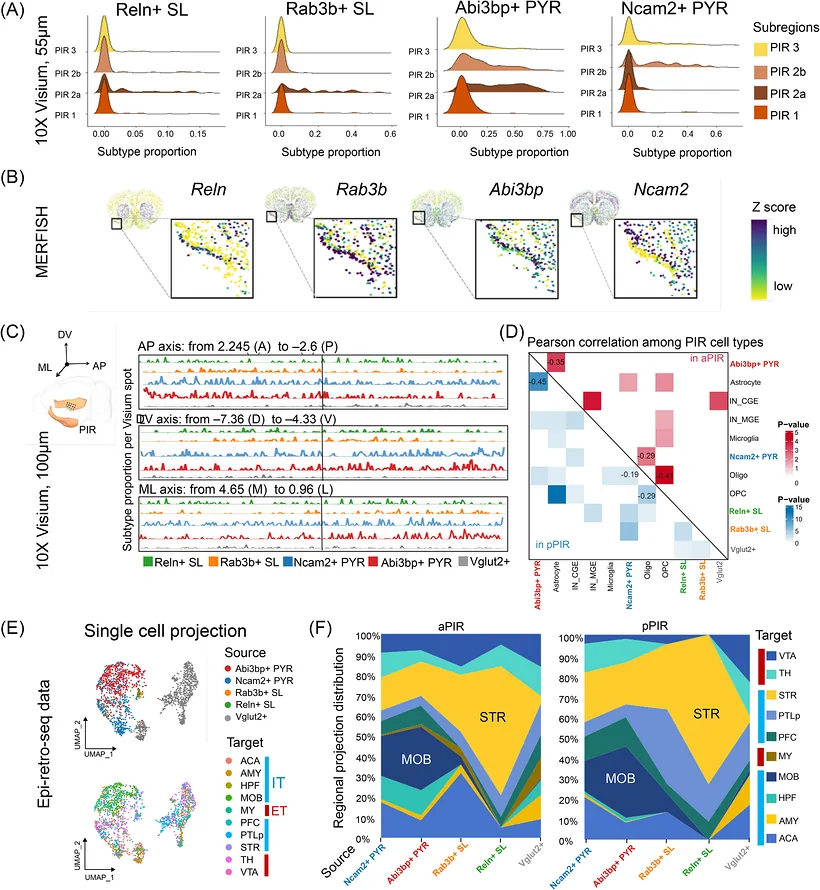
Spatial organization and projection targets of PIR PN subtypes. (A) Ridge plots showing estimated PN subtype proportions obtained by deconvolution across PIR subregions. (B) Spatial distribution of PN subtype marker expression in a coronal section of the aPIR from a published MERFISH dataset.^35^ (C) Line plots of subtype proportions along anatomical gradients, derived from a whole‐brain spatial transcriptomic atlas.^37^ Background grid lines indicate 10% intervals of estimated cell‐type proportion. Anatomical gradients include anterior‐posterior (AP), medial‐lateral (ML), and dorsal‐ventral (DV) axes. N = 1901 spatial spots. (D) Pearson correlation coefficient heatmaps representing the spatial co‐localization of each PN subtype with major glial and neuronal cell types within individual spatial spots in the aPIR and pPIR. IN_CGE, caudal ganglionic eminence‐derived interneurons; IN_MGE, medial ganglionic eminence‐derived interneurons; Oligo, oligodendrocytes; OPC, oligodendrocyte precursor cells. (E) UMAP visualization of PN subtypes from a single‐neuron projection‐mapping dataset (Epi‐retro‐seq),^70^ coloured by their major projection targets. N = 1981 single neurons. F: Stacked area plots showing the relative contribution of four PN subtypes from the aPIR and the pPIR to projections targeting ten brain regions. IT, intratelencephalic; ET, extratelencephalic. AMY, amygdala; HPF, hippocampal formation; ACA, anterior cingulate area; MOB, main olfactory bulb; PFC, prefrontal cortex; PTLβ, posterior parietal association area layer β; STR, striatum; TH, thalamus; VTA, ventral tegmental area; MY, medulla.

Spatial mapping of neuronal subtypes can reveal their anatomical organization.^51^, ^52^ Using a high‐resolution spatial transcriptomic dataset of the aPIR generated in our previous study,^53^ we characterized the spatial distribution of molecularly defined PN subtypes. This analysis revealed distinct laminar preferences among PN subtypes. Abi3bp+ PYR neurons occupied both layers IIa and IIb, whereas Ncam2+ PYR neurons were preferentially localized to layer IIb adjacent to layer III (Figure 2A). In contrast, Reln+ and Rab3b+ SL neurons were mainly enriched in layer IIa. Consistent with our spatial mapping results, the corresponding subtype marker genes showed similar laminar expression patterns in two independent spatial transcriptomic datasets based on MERFISH^35^ (Figure 2B) and Stereo‐seq (Figure S2E).

Beyond laminar organization, we next investigated whether PN subtypes also exhibit regional spatial preferences across the entire PIR. Using a whole‐brain spatial transcriptomic atlas,^37^ we deconvolved PN subtype distributions across PIR regions based on subtype‐specific signatures. All PN subtypes were broadly distributed along the dorsal‐ventral axis (Figure 2C). Despite this broad distribution, Rab3b+ SL and Abi3bp+ PYR neurons were preferentially enriched in posterior PIR regions, and Rab3b+ SL neurons showed an additional medial‐lateral distribution bias.

#### 2.1.4 Distinct cellular context and projection targets of PN subtypes

Neuronal identity is shaped by molecular expression, the local cellular environment and connectivity patterns.^48^ To explore whether PN subtypes are associated with distinct local cellular environments, we analysed cell‐type spatial associations based on spatial deconvolution of our spatial transcriptomic dataset.^53^ Correlation analysis of cell‐type proportions across spatial spots revealed distinct relationships between PN subtypes and glial populations (Figure 2D). Abi3bp+ PYRs showed a negative spatial association with astrocytes, while Ncam2+ PYRs showed a negative correlation with oligodendrocytes.

We next examined whether transcriptomically defined PN subtypes differ in their projection targets by reanalysing an epi‐retro‐seq dataset,^38^ which links epigenomic profiles of individual neurons with their projection targets. Using subtype‐associated signatures identified from our snRNA‐seq analysis, we assigned glutamatergic neurons to four molecular groups corresponding to the PN subtypes (Figure 2E, Figure S2F). Projection analysis revealed distinct target distributions among these molecular groups (Figure 2F). Compared with PYR neurons, SL neurons showed preferential association with striatal targets, whereas PYR neurons were more associated with main olfactory bulb projections. These findings support the correspondence between transcriptomic identity and projection organization of PN subtypes.

### 2.2 EC layer II‐like Reln+ SL population is selectively vulnerable in AD

**FIGURE 3:**
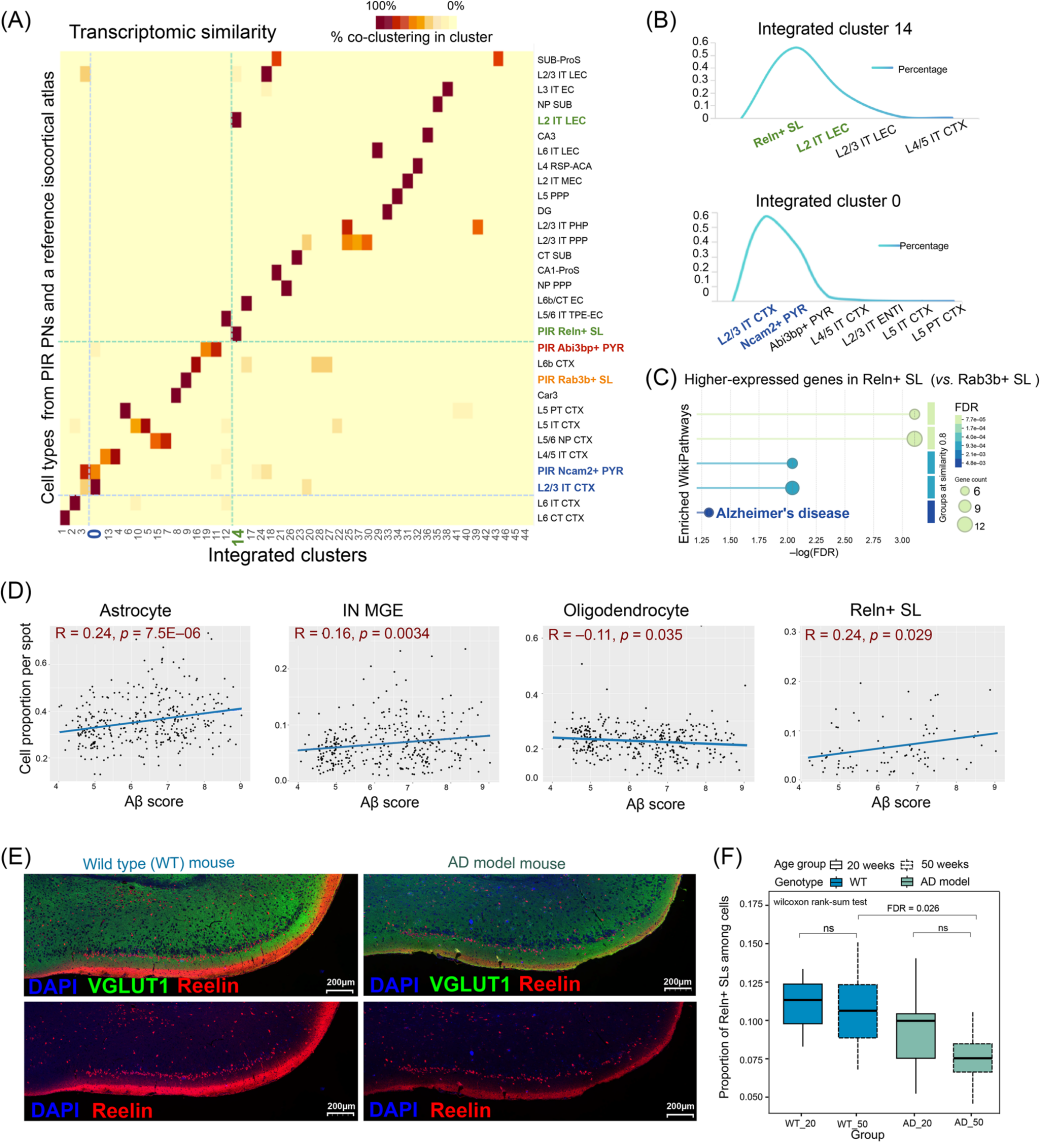
The Reln+ SL is vulnerable to Aβ accumulation in AD mouse models. (A) Co‐clustering heatmap from the integration of piriform cortex neurons^33^ with a reference mouse brain atlas^39^ (Yao 2021) using canonical correlation analysis. Each value in the heatmap represents the percentage of a given neuronal population (rows) that co‐clusters within a specific integrated cluster (columns), calculated as the proportion of cells from that population assigned to the cluster, divided by the population's total cell count across all clusters, and multiplied by 100%. LEC, lateral entorhinal cortex; MEC, medial entorhinal cortex. (B) Composition of key integrated clusters identified in (A), demonstrating selective molecular correspondence: integrated cluster 14 is enriched for PIR Reln+ SL and EC layer II excitatory neurons (L2 IT LEC), while integrated cluster 0 is dominated by PIR Ncam2+ PYR and neocortical layer II/III IT projection neurons (L2/3 IT CTX). IT: intratelencephalic. (C) Enrichment analysis of WikiPathways for genes upregulated in Reln+ SL subtype compared to the Rab3b+ SL subtype. (D) Scatter plots showing spatial correlations between deconvoluted cell type abundance (*y*‐axis) and Aβ plaque density (*x*‐axis) for each Visium spot in an AD mouse model. Only cell types with statistically significant correlations (*p* < .05) are displayed, with their Pearson correlation coefficient (R) and *p*‐value annotated. Aβ: Amyloid‐beta. IN MGE: Interneuron derived from Medial Ganglionic Eminence. (E) Representative multiplex immunofluorescence images of the pPIR from 50‐week‐old WT and AD model mice. For each genotype, the top panel shows Reelin (red) co‐localized with the glutamatergic neuronal marker VGLUT1 (green), and the bottom panel shows the Reelin channel alone. Nuclei are counterstained with DAPI (blue). Scale bar: 200 µm (applies to all fields). WT: wild‐type; AD: Alzheimer's disease. (F) Grouped box plot comparing the proportions of Reln+ SL neurons between WT and AD genotypes across two age groups (20 and 50 weeks).

Recent studies have shown that the EC is affected early in AD, with selective vulnerability of layer II excitatory neurons.^54^ We investigated whether specific PIR PN subtypes show transcriptomic similarity to AD‐vulnerable cortical populations. We integrated our PIR PN snRNA‐seq dataset^33^ with a mouse brain isocortical atlas^39^ using CCA. Joint clustering showed that Reln+ SL neurons co‐clustered with layer II lateral entorhinal area neurons (L2 ENTl), with 94.8% of Reln+ SL neurons and 98.6% of L2 ENTl assigned to the same integrated cluster (Figure 3A,B). In contrast, Ncam2+ PYRs preferentially co‐clustered with neocortical L2/3 excitatory neurons, whereas Rab3b+ SLs co‐clustered with EC layer III excitatory neurons (Figure 3B; Figure S3A). Consistent with the integration results, snRNA‐seq expression profiles further supported the molecular similarity between PIR PN subtypes and their co‐clustering cortical populations (Figure S3B). Reln+ SLs and Rab3b+ SLs expressed molecular signatures of EC layer II and III neurons,^1^ respectively, whereas Ncam2+ PYRs showed features of neocortical LII/III neurons.^55^ Moreover, genes upregulated in Reln+ SL neurons relative to Rab3b+ SL neurons were enriched for Alzheimer's disease‐related pathways (Figure 3C).

These findings suggest that Reln+ SL neurons represent an EC layer II‐like excitatory population in the PIR, raising the possibility of its selective vulnerability in AD.

#### 2.2.1 Reln+ SL abundance is spatially associated with Aβ pathology in AD

Aβ deposition is a pathological hallmark of AD.^56^ We analysed a spatial transcriptomic dataset^43^ from AD mouse models, in which each spatial spot was annotated with Aβ plaque density. We estimated the relative abundance of Reln+ SLs in each spatial spot using deconvolution and examined its association with local Aβ burden. Reln+ SL abundance showed a significant positive correlation with Aβ burden (*R* = .24, *p* = .029, Figure 3D). Similar positive correlations were observed for astrocytes and medial ganglionic eminence (MGE)‐derived interneurons, whereas oligodendrocytes showed a negative correlation. These associations are consistent with established AD‐related cellular changes, including reactive astrogliosis,^57^ oligodendrocyte dysfunction and loss,^58^ and selective vulnerability of inhibitory neuron subtypes (e.g., SST+ MGE neurons) in the PIR.^27^ Multiplex immunofluorescence staining for Aβ, Reelin and DAPI in PIR sections from 5XFAD AD model mice showed that Reelin+ excitatory neurons were present in Aβ plaque‐containing regions (Figure S3C). Similarly, GFAP immunostaining captured astrocytic processes surrounding Aβ plaques (Figure S3D). These histological observations provided additional support for the spatial association identified by transcriptomic analysis.

We next quantified the abundance of Reln+ SL neurons in the PIR of wild‐type (WT) and AD model mice using multiplex immunofluorescence staining. The abundance of Reln+ SL neurons was decreased in AD mice, with a significant reduction observed in the PIR at 50 weeks compared with WT mice (Figure 3E,F). Together with their spatial association with Aβ pathology, these findings support the possibility that Reln+ SLs are vulnerable to AD‐related pathology.

#### 2.2.2 Cellular microenvironment associated with Reln+ SLs in AD models

**FIGURE 4:**
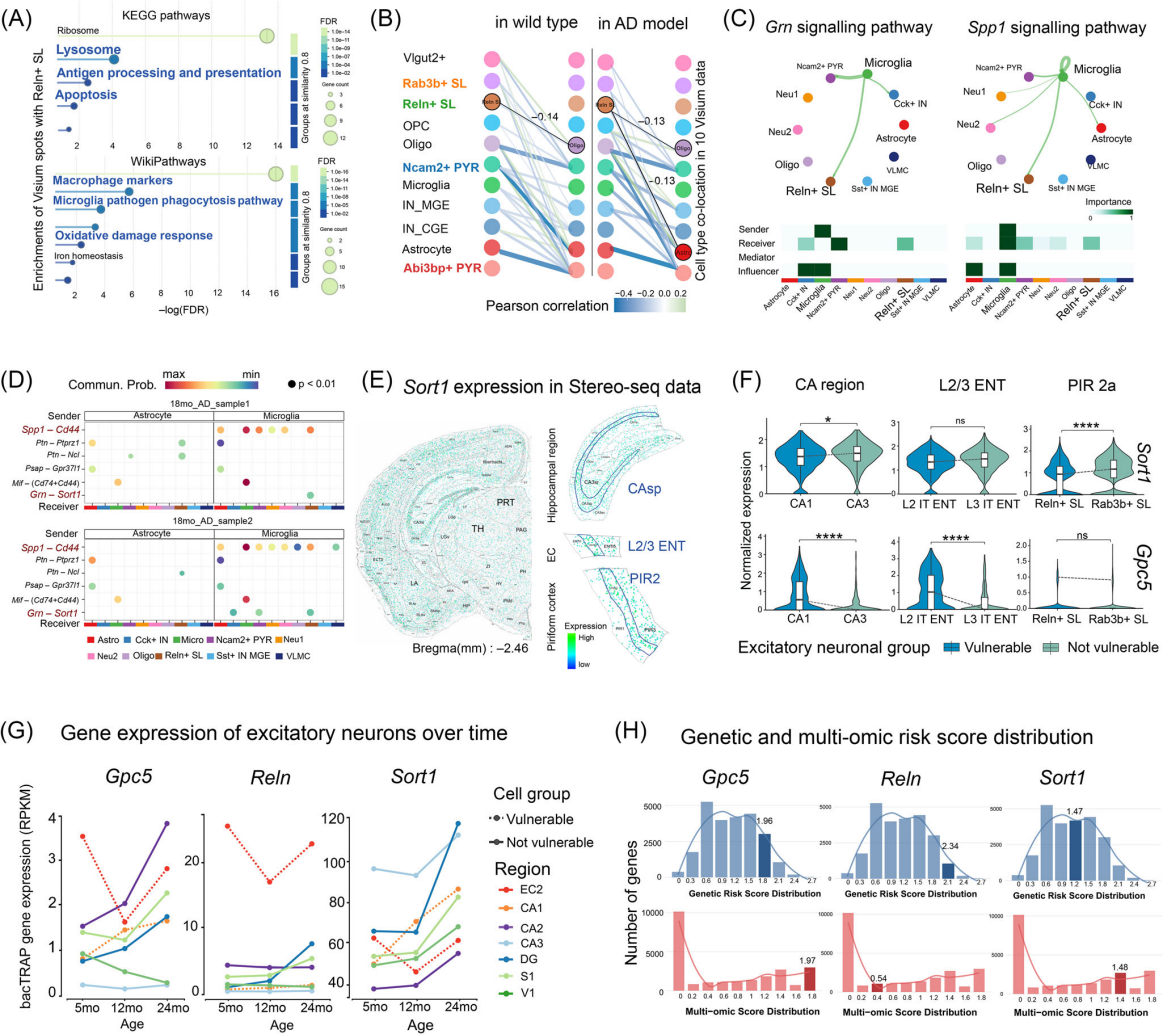
Molecular and microenvironmental features associated with the selective vulnerability of the Reln+ SL subtype. (A) Pathway enrichment analysis of spatial regions with high Reln+ SL abundance. KEGG (top) and WikiPathways (bottom) pathway enrichment analyses were performed on genes upregulated in Visium spots with high Reln+ SL deconvolution‐estimated proportions (top 30%) compared with all other spots from the AD mouse Visium dataset.^43^ (B) Comparative analysis of cellular microenvironments. Network plot showing pairwise spatial correlations between major cell types in WT and AD mice. Edges indicate significant correlations (*p* < 0.05), with edge width representing correlation strength and colour indicating direction (green: positive correlation; blue: negative correlation). Correlations involving Reln+ SLs are highlighted with black edges. (C) Altered intercellular signalling pathways in AD olfactory cortex (Stereo‐seq data^40^). Bubble plot showing significantly altered secretory signalling pathways in 18‐month‐old WT vs. AD mice. Heatmaps show the contribution of sending and receiving cell types to selected pathways. VLMC, vascular leptomeningeal cells. (D) Dot plot quantifying the interaction strength of key ligand‐receptor pairs within the altered signalling pathways identified in (C). Commun. prob, inferred communication probability. (E) Spatial distribution of *Sort1* expression in a coronal section of the WT mouse brain (Stereo‐seq data^36^). CAsp, Cornu Ammonis superficial layer. L2/3 ENT, layer 2/3 entorhinal cortex neurons. (F) Expression of *Sort1* and *Gpc5* in glutamatergic neurons across three brain regions using published snRNA‐seq datasets. Vulnerable‐resilient pairs included hippocampal CA1 vs. CA3, L2 IT ENT vs. L3 IT ENT, and PIR Reln+ SL vs. Rab3b+ SL neurons. EC: entorhinal cortex; CA: Cornu Ammonis. G: Age‐associated expression dynamics of *Gpc5, Sort1*, and *Reln* in vulnerable neuronal subpopulations during aging (5, 12 and 24 months) in WT mice, as profiled by bacTRAP snRNA‐seq data.^41^ (H) Overall distribution of genetic and multi‐omics risk scores for 24 395 protein‐coding genes. The positions of *GPC5*, *RELN*, and *SORT1* are highlighted with their corresponding risk scores.

Selective vulnerability in AD may arise from extrinsic microenvironmental stress or intrinsic molecular programs.^3^ To characterize the cellular microenvironment associated with Reln+ SLs, we analysed a publicly available 10x Visium dataset^43^ from AD mouse models. Within the AD samples, genes enriched in Reln+ SL‐high spots (top 30% of deconvoluted Reln+ SL abundance) were identified by comparison with the remaining spots. Functional enrichment analysis of these genes showed significant enrichment of ribosome‐ and lysosome‐related pathways in KEGG, as well as pathways associated with macrophage activation and oxidative stress in WikiPathways (Figure 4A). Moreover, Reln+ SL abundance was negatively correlated with astrocyte abundance across spatial spots in AD, but not in WT mice (Figure 4B), suggesting an AD‐specific alteration in the spatial relationship between these cell populations.

To investigate whether these microenvironmental alterations were accompanied by changes in intercellular signalling, we analysed a high‐resolution Stereo‐seq dataset from AD models.^40^ Cell‐cell communication analysis based on ligand‐receptor interaction inference identified increased *Grn*–*Sort1* and *Spp1*–*Cd44* signalling interactions in AD but not in age‐matched WT mice (Figure 4C). Microglia were identified as the predominant sender population expressing *Grn* and *Spp1*, whereas Reln+ SL neurons were predicted as receiver populations expressing *Sort1* and *Cd44*, respectively (Figure 4D).

Together, these analyses identify an AD‐associated microenvironment surrounding Reln+ SL neurons, characterized by altered immune‐related intercellular signalling, providing potential molecular features associated with Reln+ SL vulnerability.

### 2.3 Molecular profiles associated with Reln+ SL vulnerability in AD

#### 2.3.1 Shared *Gpc5*‐high and *Sort1*‐low expression of Reln+ layer II neurons in the normal piriform and entorhinal cortex

To identify molecular features associated with selective vulnerability, we examined whether vulnerable neuronal populations share transcriptional signatures across PIR and EC. Because *Grn*–*Sort1* signalling interaction was specifically increased in the AD PIR, we first investigated the spatial distribution of *Sort1* expression using a high‐resolution Stereo‐seq dataset^36^ from the WT mouse brain. *Sort1* expression was enriched in specific neuronal laminae harbouring selectively vulnerable populations, including hippocampal CA1 and EC layers II neurons previously implicated in AD vulnerability,^3^, ^59^ as well as the piriform layer II neurons identified in our study (Figure 4E).

We compared gene expression difference across established vulnerable‐resilient excitatory neuronal subtype pairs in the normal mouse brain,^33^, ^39^ including hippocampal CA1 versus CA3 neurons, EC layer II versus III neurons, and PIR Reln+ versus Rab3b+ SLs. Vulnerable neurons showed higher *Gpc5* and lower *Sort1* expression relative to their resilient counterparts (Figure 4F). Unlike hippocampal vulnerable neurons, vulnerable layer II neurons in EC and PIR were characterized by high *Reln* expression (Figure S4A). Analysis of a published human single‐cell transcriptomic atlas from cognitively normal individuals^9^ further revealed conserved *GPC5*‐high and *SORT1*‐low expression patterns in the EC (Figure S4B).

#### 2.3.2 *Gpc5* and *Sort1* expression in vulnerable neurons during normal aging in mice

Given that ageing is a risk factor for AD, we next investigated the age‐dependent dynamics of *Gpc5* and *Sort1* expression in vulnerable and resilient neuronal populations. We analysed a mouse ageing snRNA‐seq dataset of glutamatergic neurons at three time points (5, 12 and 24 months) across the EC, hippocampus, and neocortex.^41^ Although all neuronal populations exhibited age‐associated transcriptional changes, vulnerable neurons exhibited different trajectories of *Gpc5* and *Sort1* expression (Figure 4G).

Resilient neurons showed a progressive increase in *Sort1* expression, with a greater increase occurring between 12 and 24 months than between 5 and 12 months. In contrast, vulnerable CA1 neurons showed a faster increase in Sort1 expression between 5 and 12 months, followed by a slower increase between 12 and 24 months. Notably, EC layer II Reln+ neurons exhibited reduced *Gpc5* and *Sort1* expression from 5 to 12 months, followed by increased expression at 24 months. This reduction‐and‐recovery dynamics was specific to the EC layer II Reln+ neurons and was not observed in CA1 neurons.

#### 2.3.3 *GPC5* and *SORT1* expression in RELN+ EC layer II neurons during AD Braak stages in human samples

**FIGURE 5:**
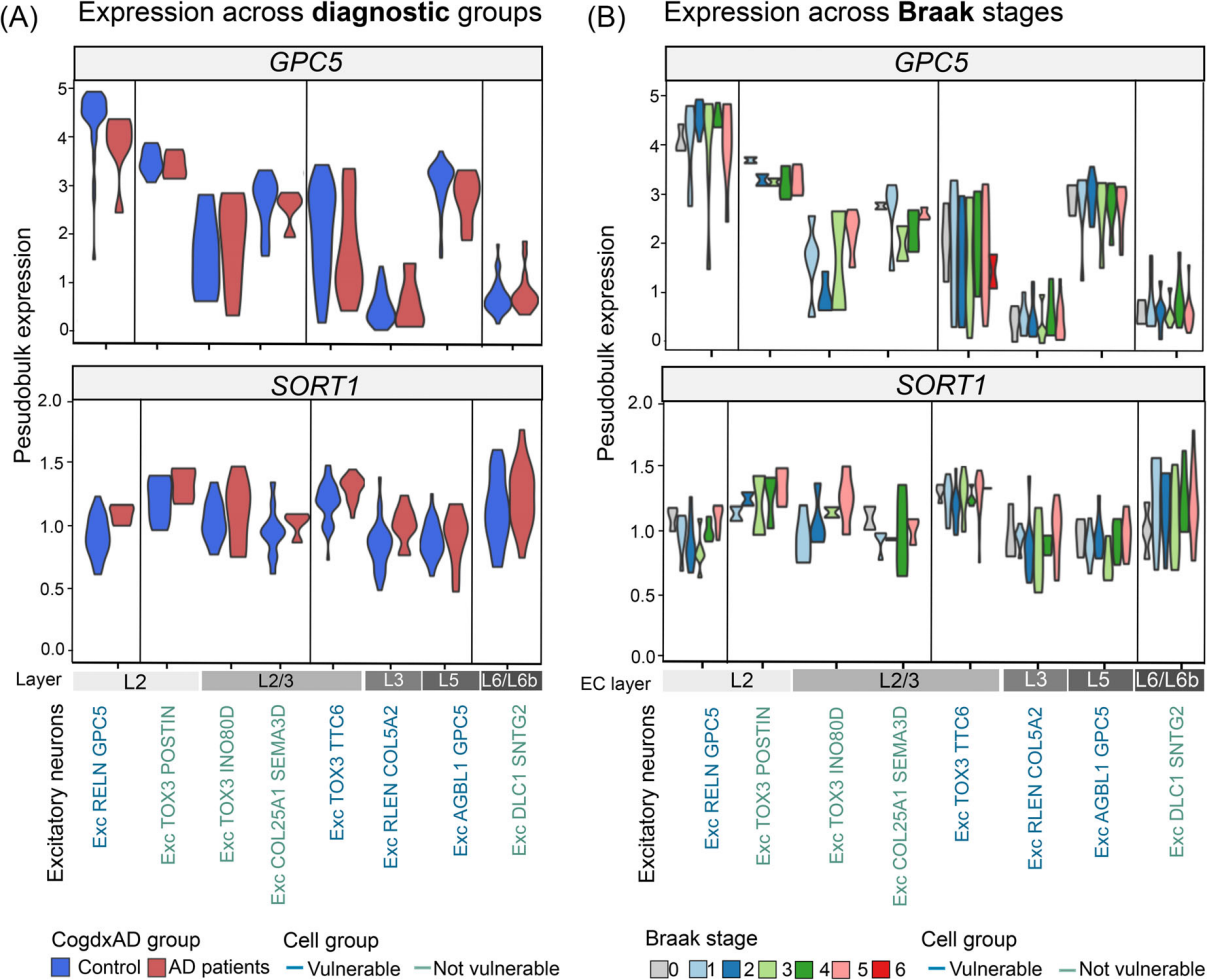
Expression patterns of *GPC5* and *SORT1* across Braak stages in human AD. (A) Cross‐sectional expression profile across diagnostic groups. Violin plots show the expression of *GPC5* (top) and *SORT1* (bottom) in excitatory neuron subtypes of the EC. Expression values represent the average expression of each neuronal subtype within individual samples. Samples are grouped by CogdxAD clinical diagnosis (cognitively normal or AD). Subtypes are ordered by laminar position (L2, L2/3, L3, L5 and L6/L6b). (B) Expression patterns across Braak stages in human AD. Violin plots show pseudobulk expression of *GPC5* (top) and *SORT1* (bottom) for each excitatory neuron subtype. Within each subtype group, separate violins represent the distribution across Braak stages I–VI (left to right).

The Braak staging system^4^ describes the spatial progression of tau tangles across different brain regions and cell types. To characterize the progression‐dependent dynamics of *GPC5* and *SORT1* expression during AD, we re‐analysed a recently published public single‐cell AD atlas,^9^ which included 283 postmortem brain samples from 48 cognitively normal individuals and AD patients across Braak stages I–VI. In cognitively normal controls, RELN+ GPC5+ excitatory neurons in EC layer II displayed the highest *GPC5* and lowest *SORT1* expression among all EC neuronal populations (Figure 5A). When comparing clinically diagnosed AD patients with cognitively normal controls, *GPC5* expression selectively decreased in layer II RELN+GPC5+ neurons, whereas it remained relatively stable in other vulnerable populations. In contrast, *SORT1* expression increased broadly across vulnerable neuronal populations; however, EC layer II RELN+GPC5+ neurons retained comparatively low *SORT1* expression.

Braak stages I–IV generally correspond to early pathological progression, which may occur before or around the onset of clinical symptoms,^4^ whereas stages V–VI are associated with more advanced disease. During early AD progression (Braak stages I‐IV), EC layer II RELN+GPC5+ neurons showed increased *GPC5* and decreased *SORT*1 expression (Figure 5B). This expression was not observed in other vulnerable neuronal populations within the EC, such as TOX3+TTC6+ neurons in layers II/III.

#### 2.3.4 *Gpc5* and *Sort1* expression within EC layer II neurons in mouse brains

Within the EC, the LEC exhibits earlier and more pronounced atrophy than the MEC during AD progression.^4^ ^.^ ^7^ Having identified *GPC5* and *SORT1* as vulnerability‐associated molecular features of EC layer II Reln+ neurons, we next asked whether molecular programs associated with these genes differ between LEC and MEC layer II neurons and may reveal characteristics associated with regional vulnerability.

**FIGURE 6:**
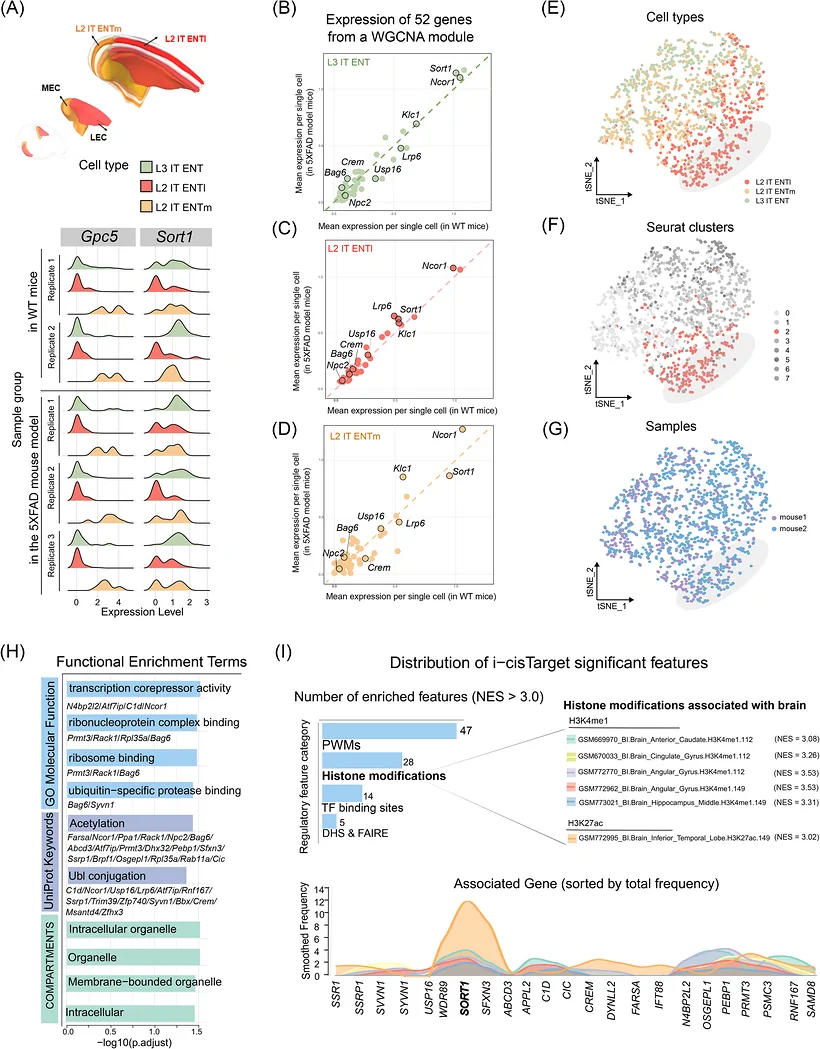
A WGCNA‐derived gene module distinguishing layer II excitatory neurons in the lateral and medial entorhinal cortex. (A) Schematic representation of the analysed L2 ENT populations (top) and expression profiles of *Gpc5* and *Sort1* across individual samples from WT and AD mouse models. Ridge plots show gene expression distributions across L2 IT ENT, L2 IT ENTm and L3 IT ENT neuronal populations. ENTl, lateral entorhinal cortex; ENTm, medial entorhinal cortex. (B–D): Scatter plots comparing mean gene expression between WT and AD mice for genes within the WGCNA‐derived 52‐gene module across ENT excitatory neuronal populations. Each dot represents one gene, with WT and AD expression shown on the *x*‐ and *y*‐axes, respectively. L3 IT ENT (B), L2 IT ENTl (C), and L2 IT ENTm (D) populations are shown. Selected module genes are highlighted. E‐G: Validation of the 52‐gene molecular module using independent reference dataset^39^ (Yao 2021). t‐SNE visualization of ENT excitatory neurons based on the 52‐gene module expression profile, coloured by original cell‐type annotations (E) or Seurat‐derived unsupervised clusters (F) or samples (G). (H) Functional enrichment analysis of genes within the 52‐gene module. Gene Ontology terms are shown based on enrichment significance. (I) i‐cisTarget analysis identifying enriched transcription factor binding motifs and associated regulatory features within the 52‐gene module. Predicted regulatory factors and associated genomic features are displayed. NES, normalized enrichment score; PWMs, position weight matrices; DHS, DNase I hypersensitive sites; FAIRE, Formaldehyde‐Assisted Isolation of Regulatory Elements.

We first re‐analysed a published snRNA‐seq dataset from WT and AD mouse brains,^60^ focusing on L2 ENTl, L2 ENTm and L3 ENT excitatory neurons. The expression patterns of *Reln*, *Gpc5* and *Sort1* were examined across these populations (Figure 6A). Both L2 ENTl and L2 ENTm neurons were characterized by high *Reln* expression, with lower *Sort1* expression compared with L3 ENT neurons (*Sort1*: log2FC = −0.60, FDR = 5.64 × 10^−3^). Within EC layer II neurons, *Sort1* levels were comparable between ENTl and ENTm, with only a modest reduction in ENTl neurons (log2FC = −0.47, not significant). In contrast, *Gpc5* expression was higher in L2 ENTm than L2 ENTl neurons (log2FC = 4.27, FDR = 4.5 × 10^−^^14^). Despite these distinct expression patterns, single‐gene analyses alone may not fully explain the molecular heterogeneity between L2 ENTl and L2 ENTm neurons. We therefore extended our analysis to co‐expression networks to identify coordinated gene modules and their associated biological processes.

#### 2.3.5 A *Sort1*‐containing module separates lateral from medial populations within EC layer II

To identify gene modules associated with regional differences among EC layer II neuron populations, we performed WGCNA analysis separately for L2 ENTl, L2 ENTm and L3 ENT neurons from WT and AD mice. We identified a 52‐gene co‐expression module specifically enriched in ENTl neurons (Figure 6B). Notably, this module contained *Sort1* and a set of genes exhibiting coordinated regulation in ENTl neurons. Compared with WT mice, genes within this module displayed a consistent upregulation pattern in AD ENTl neurons, with the majority showing increased expression (binomial test, FDR = 0.003). In contrast, these genes exhibited no consistent directional changes in L2 ENTm or L3 ENT neurons (Figure 6 C,D).

To determine whether this 52‐gene module represented an intrinsic molecular feature of ENTl neurons rather than an AD‐associated transcriptional response, we evaluated its ability to classify EC layer II neuronal populations in an independent snRNA‐seq atlas of the normal mouse brain.^39^ Expression of genes within this module robustly separated L2 ENTl neurons from L2 ENTm and L3 ENT neurons in PCA space, whereas L2 ENTm and L3 ENT neurons showed substantial overlap (Figure 6E–G; Figure S4D). This separation was not reproduced using randomly selected gene sets of the same size (Figure S4E), supporting the specificity of the 52‐gene module in capturing the molecular characteristics of L2 ENTl neurons.

#### 2.3.6 The *Sort1*‐containing module is enriched for proteostasis‐related processes

Functional enrichment analysis of this 52‐gene module using the Gene Ontology database revealed significant enrichment for molecular functions related to protein homeostasis (Figure 6H). Enriched terms included ubiquitin‐specific protease binding, ribonucleoprotein‐and ribosome‐associated functions, as well as transcriptional regulation.

We further characterized cis‐regulatory signatures of 52‐gene module using i‐cisTarget enrichment analysis. We identified 94 significantly enriched cis‐regulatory features (NES > 3.0), including 14 TF binding sites and 28 histone modification‐associated regions (Figure 6I). Several enriched histone modification profiles corresponded to human brain regions implicated in AD,^3^, ^61^, ^62^ including the hippocampus, angular gyrus, cingulate gyrus, and inferior temporal cortex, with enhancer‐associated marks such as H3K4me1 and H3K27ac represented. Notably, *SORT1* was repeatedly identified among genes associated with these brain‐derived regulatory signatures, highlighting its relevance within this ENTl‐associated gene module.

## 3 CONCLUSION

Selective neuronal vulnerability is a hallmark of AD, yet the intrinsic molecular features associated with the selective susceptibility of distinct neuronal populations remain incompletely understood. In this study, we performed an integrative single‐cell multi‐omic analysis to define the molecular and spatial organization of PIR PNs and identify a previously unrecognized AD‐associated vulnerable neuronal population in the PIR. We demonstrated that Reln+ PNs in the PIR exhibit a spatial association with Aβ pathology in AD models. We also identified a shared *Gpc5‐*high/*Sort1*‐low molecular signature associated with vulnerable layer II neurons across PIR and EC. Furthermore, we identified a *Sort1*‐containing co‐expression program that differentiated LEC from MEC layer II neurons and was enriched for proteostasis‐related processes, providing insights into regional differences in AD vulnerability. Previous studies have identified *Gpc5*, a cell‐surface heparan sulphate proteoglycan implicated in growth‐factor signalling and synaptic stability,^63^ as a molecular feature associated with selectively vulnerable neuronal populations.^56^, ^64^ Our study extends these observations by identifying a complementary molecular dimension involving a Sort1‐associated gene module.

Several hypotheses have been proposed concerning the intrinsic cellular features associated with selective neuronal vulnerability in AD.^3^ Among these, alterations in protein homeostasis, including protein folding, intracellular trafficking and degradation pathways, have emerged as candidate features associated with neuronal susceptibility. Although individual proteostasis factors have been characterized in the context of AD‐associated protein aggregation, their contribution to selective neuronal vulnerability remains incompletely understood. Notably, *SORT1*, an AD‐associated risk gene,^65^, ^66^ encodes a sorting receptor involved in intracellular trafficking and pathways related to protein homeostasis,^67^ processes increasingly implicated in neuronal vulnerability in AD. Consistent with this concept, a SORT1‐associated gene module we identified in LEC layer II neurons was enriched for proteostasis‐related processes, including ubiquitin‐associated regulation and ribosome‐related functions. Importantly, this molecular program distinguished LEC layer II neurons from MEC and layer III populations, suggesting that regional vulnerability may not be fully explained by individual marker genes alone but by coordinated transcriptional networks. Given that LEC undergoes earlier and more severe degeneration than MEC during AD progression, these findings are consistent with the emerging hypothesis that differences in proteostasis‐related programs may be associated with selective neuronal susceptibility in AD.

Beyond molecular characterization, we further examined the relevance of *GPC5* and *SORT1* to human AD risk using population‐level genetic and multi‐omic risk analyses (Figure 4H). Although common genetic variation in *GPC5* and *SORT1* showed limited association with AD risk, both genes exhibited significant multi‐omic risk signals (*GPC5*: *p* < 1 × 10^−6^; *SORT1*: *p* = 0Bc.00113), suggesting that their contribution to disease susceptibility may be more strongly associated with regulatory molecular alterations rather than inherited variation alone. These findings further support the potential relevance of *GPC5*/*SORT1*‐associated molecular states in human AD.

Several limitations should be acknowledged. First, our omics analyses identified associations rather than causal relationships. Future studies using cell‐type‐specific genetic manipulation will be required to determine whether *Gpc5*, *Sort1*, or their associated gene modules contribute to vulnerability. Second, the cross‐species relevance of our findings remains to be further established. Our spatial transcriptomic analysis linking Reln+ SL neurons to AD pathology was performed in mouse models driven by Aβ deposition, whereas human AD progression is characterized by the spatiotemporal spread of tau pathology. Therefore, the reduction of Reln+ SL neurons observed in mouse models should be interpreted within the context of Aβ‐associated pathology and may not fully capture the complexity of human AD progression. Despite this limitation, the conservation of the *Gpc5*/*Sort1*‐associated vulnerability signature across mouse and human single‐cell transcriptomic datasets supports the relevance of these molecular features to human AD vulnerability. Finally, our study defines molecular correlates of vulnerability but does not directly assess their impact on olfactory circuit function. Future integration of molecular profiling with functional approaches will be necessary to determine how these vulnerable neuronal states influence circuit dysfunction during AD progression.

## 4 MATERIALS AND METHODS

### 4.1 Mice and multi‐omics datasets

#### 4.1.1 Mice

All experimental animal procedures and animal care were approved by the Animal Ethics Committee of Shanghai Institute of Immunity and Infection (Approval No. A2023007). Two adults male 5XFAD C57BL/6 mice were purchased from Cyagen Biosciences Inc., and two adult male C57BL/6 wild‐type mice were obtained from Charles River Laboratories (Table S2). For each genotype, mice were assigned to two age groups (20 and 50 weeks). All animals were housed in a temperature‐ and humidity‐controlled environment under a 12‐h light‐dark cycle, with food and water provided ad libitum. Mice were used for immunofluorescence staining.

#### 4.1.2 Public datasets

To characterize the molecular diversity and AD vulnerability of PIR PNs, we integrated publicly available single‐cell multi‐omics datasets^9^, ^33^, ^34^, ^35^, ^36^, ^37^, ^38^, ^39^, ^40^, ^41^, ^42^, ^43^, ^44^, ^45^, ^47^, ^53^, ^60^ (Table S1). We first identified four transcriptional PN subtypes using a published mouse PIR snRNA‐seq dataset^33^ and validated these classifications using independent epigenomic atlases.^34^, ^35^, ^42^ We next mapped these subtypes onto their anatomical locations using high‐resolution spatial transcriptomic resources^35^, ^36^, ^37^, ^42^, ^53^ and profiled their projection targets with an epi‐retro‐seq dataset.^38^ Finally, we investigated subtype‐specific vulnerability in AD models^40^, ^43^ and identified vulnerability‐associated molecular profiles in a mouse cortical reference atlas,^39^ normal ageing time‐course data,^41^ and a human Braak‐staged AD atlas.^9^

The samples included mouse models of Alzheimer's disease (wild type, 5XFAD, App^NL‐G‐F^; 6 weeks to 18 months of age) and human post‐mortem tissues from patients and controls with Braak staging. The datasets cover seven omics technologies, including single‐nucleus RNA sequencing (snRNA‐seq), single‐nucleus assay for transposase‐accessible chromatin sequencing (snATAC‐seq), single nucleus methylcytosine sequencing (snmC‐seq2), epigenomic retrograde sequencing (epi‐retro‐seq), 10X Genomics spatial gene expression (10x Spatial Visium), spatial enhanced resolution omics‐sequencing (Stereo‐seq) and multiplexed error‐robust fluorescence in situ hybridization (MERFISH).

### 4.2 Tissue processing and histology

#### 4.2.1 Mouse brain perfusion, sectioning and immunofluorescence staining

After deep anaesthesia with 2,2,2‐tribromoethanol (330 mg/kg, 2.5% solution), mice were transcardially perfused sequentially with 30 mL of 0.9% normal saline preheated to 37°C, followed by 30 mL of 4% paraformaldehyde (PFA). Brains were rapidly dissected and fixed in 4% PFA for 24 h. Upon completion of fixation, the brains were embedded in paraffin, and 3 µm‐thick coronal sections were immediately prepared using an adult mouse brain slicer. The paraffin sections were subjected to sequential deparaffinization, rehydration, and antigen retrieval procedures. To reduce non‐specific antibody binding, all sections were blocked with 3% donkey serum. Target proteins were labelled using tyramide signal amplification (TSA) technology, and cell nuclei were finally counterstained with 4',6‐diamidino‐2‐phenylindole (DAPI). Fluorescence images of the stained sections were acquired via whole‐slide scanning using a KFBIO slide scanner. The primary antibodies used in this study included: anti‐vesicular glutamate transporter 1 (VGLUT1, Cat. No. 55491‐1‐AP; Proteintech), anti‐neural cell adhesion molecule 2 (NCAM2, Cat. No. 13850‐1‐AP; Proteintech), anti‐RAS‐related protein Rab‐3B (RAB3B, Cat. No. 15774‐1‐AP; Proteintech), anti‐reelin (RELN, Cat. No. 20689‐1‐AP; Proteintech), and anti‐ABI family member 3 binding protein (ABI3BP, Cat. No. PC6506S; Abmart), and DAPI (Cat. No. C0060; Solarbio). The secondary antibodies were obtained from a Five‐plex Multiplex Immunofluorescence Kit.

To examine the spatial relationship between Aβ pathology, Reln‐positive neurons, and astrocytes, multiplex immunofluorescence staining was performed using the TSA‐based workflow described above. Briefly, sections were incubated overnight at 4°C with a primary antibody cocktail containing antibodies against Reelin, GFAP, and Aβ (anti‐B‐A,6E10, Cat. No. 803004; BioLegend), followed by sequential fluorescence amplification using TYR‐555, TYR‐651 and TYR‐488 fluorophores, respectively. After DAPI nuclear counterstaining and mounting with anti‐fade medium, multi‐channel fluorescence images were acquired for co‐localization analysis.

#### 4.2.2 Public histology resources

**ISH data** were obtained from the Allen Brain Atlas (https://mouse.brain‐map.org/)^68^ to examine the expression patterns of markers specific to PN subtypes. ISH images for several markers, including *Ntng1, Reln, Rab3b, Slc30a3* and *Ncam2*, were retrieved, as these genes were available in the Atlas and identified as characteristic of distinct PN subtypes in our analyses. Data were sourced from coronal brain sections of the aPIR region.

**Immunofluorescence staining data** for *Ncam2* and *Slc17a7* in the WT mouse PIR were obtained from the Human Protein Atlas (https://www.proteinatlas.org/ENSG00000154654‐NCAM2/brain, https://www.proteinatlas.org/ENSG00000104888‐SLC17A7/brain).^18^ Protein expression was examined in coronal sections at bregma +0.14 and bregma +1.18, with the three PIR sublayers delineated as indicated by the atlas.

#### 4.2.3 Quantification of immunostaining images

For each brain, two coronal sections were analysed, corresponding to the anterior and posterior PIR regions at approximately bregma positions +0.02 mm and –1.06 mm, respectively. Immunostaining images were exported to Fiji for cell‐based quantification. Layer II of the PIR was manually defined as the region of interest (ROI). DAPI staining was used to visualize nuclei, and VGLUT1‐positive neurons were identified as excitatory neurons. Reln+ SL neurons were defined as Reelin+/VGLUT1+ double‐positive neurons. The proportion of Reln+ SL neurons was calculated relative to the total number of VGLUT1‐positive excitatory neurons within each ROI. All image acquisition and quantification procedures were performed by researchers blinded to animal genotype.

### 4.3 Data integration and harmonization across multi‐omics datasets

To enable consistent comparison across studies, integration was performed across within individual modalities and across molecular modalities, with cell‐type annotations harmonized across studies. Integration quality was assessed by evaluating dataset‐origin distributions within the resulting embeddings to determine whether clustering reflected biological variation rather than technical differences (Figure S5).

#### 4.3.1 Integration of multiple snRNA‐seq datasets

**Multiple PIR snRNA‐seq samples from the same study** (Zeppilli)^33^ were integrated to correct for sample‐associated batch effects. Batch correction was performed using Harmony^69^ with default parameters, and the Harmony‐corrected embedding was used for downstream dimensionality reduction and clustering analyses (Figure S5A–C).

**For integration of snRNA‐seq datasets generated from different studies,** we incorporated publicly available reference datasets to contextualize PIR neuronal populations within established transcriptomic taxonomies. Cross‐dataset integration was performed using the Seurat canonical correlation analysis (CCA)‐based integration workflow. Each dataset was independently normalized using SCTransform, and 2000 highly variable genes were selected for integration. Dataset anchors were identified using CCA with SCT normalization based on 30 dimensions, followed by anchor‐based dataset integration. Principal component analysis (PCA) was performed on the integrated dataset, and the first 200 principal components were retained for downstream analyses. A shared nearest neighbour graph was constructed using the first 20 principal components, followed by Louvain clustering at a resolution of 0.8. The integrated dataset was visualized using uniform manifold approximation and projection (UMAP). Two published mouse atlases were used for integration with the PIR snRNA‐seq dataset.^33^ The isocortical mouse atlas^39^ (Yao 2021) was used to assess transcriptomic correspondence between PIR and other cortical neuronal populations (Figure S5D–F). The whole‐mouse‐brain atlas^47^ (Yao 2023) was used for reference‐based mapping of piriform cortical subtypes (Figure S5J).

#### 4.3.2 Reference‐based integration of snRNA‐seq and scATAC‐seq datasets

Cross‐modality datasets were integrated using Seurat's reference‐based label transfer method. The well‐annotated snRNA‐seq data^33^ were used as the ‘reference’ and the scATAC‐seq data^34^ (processed into gene activity scores) was used as the ‘query’. Cross‐modality anchors were identified using the *FindTransferAnchors* function with the CCA reduction. Cell‐type annotations from the snRNA‐seq reference dataset were transferred to scATAC‐seq cells using *TransferData* function, with the first 2–30 dimensions of the latent semantic indexing embedding used as the weight reduction for prediction. Prediction scores and transferred cell‐type labels were added to the scATAC‐seq dataset for downstream analyses (Figure S5H,I).

#### 4.3.3 Harmonization of cell‐type annotations across datasets

Cell‐type annotations across single‐cell and spatial datasets were harmonized based on a unified transcriptomic taxonomy established from the PIR snRNA‐seq reference dataset.^33^ Broad cell classes, including excitatory neurons, inhibitory neurons, glial cells and vascular cell types, were identified using established marker gene profiles from previous studies^33^ (More details in Method [Section 4.4.1]–Cell type annotation). Within excitatory neuronal populations, PIR neuronal subtypes were consistently defined based on subtype‐specific marker signatures, including *Slc17a6* for Vglut2+ neurons, *Ntng1* and *Reln* for SL subtypes, and *Slc30a3*, *Ncam2* and *Abi3bp* for PYR subtypes.

For datasets lacking direct transcriptomic annotation, including snATAC‐seq, spatial transcriptomics, and single‐neuron methylation datasets, cell‐type identities were assigned based on the PIR reference taxonomy^33^ using label transfer (as described in Section 4.3.2), reference‐based deconvolution (as described in Section 4.4.4), or marker‐based classification approaches (SCINA), respectively.

For marker‐based annotation, SCINA (v1.2.0), a semi‐supervised classification algorithm that assigns cell identities based on predefined marker gene sets, was applied to generate automated cell‐type annotations. A marker signature matrix was generated for each dataset based on the unified annotation scheme described above. Input matrices were log‐normalized counts for snRNA‐seq and Stereo‐seq data, and a negative log transformation (–log_2_(mCH+1)) for epi‐retro‐seq mCH profiles to correct for the inverse mCH‐expression relationship. SCINA was then run with default parameters (max_iter = 100, convergence = 1e–4) to generate automated cell‐type labels. This approach was applied to the PIR snRNA‐seq dataset, the epi‐retro‐seq dataset,^70^ and the Stereo‐seq AD mouse model dataset.^40^

**Annotation consistency was further evaluated** using marker expression. In the stereo‐seq dataset, subtype marker genes showed the expected expression patterns across annotated neuronal populations (Figure S3E). Similarly, scATAC‐seq and methylation‐based datasets showed concordant subtype‐associated chromatin accessibility and mCH patterns around corresponding subtype marker genes (Figure S1D, Figure S2F), further supporting the consistency of subtype annotation across modalities.

### 4.4 Definition and validation of PN subtypes

#### 4.4.1 Single‐nucleus RNA‐seq analyses

##### Pre‐processing

Data were downloaded from GSE239477.^33^ Quality control (QC) was performed in Seurat,^71^ and cells were retained if they contained 200–5000 detected features and showed < 10% mitochondrial transcripts. Doublet removal was then conducted using DoubletFinder^72^ v2.2.0 (7% per 10 000 cells). Cells derived from the agranular insular cortex (AI) and primary somatosensory cortex (SSp) were excluded so that only aPIR and pPIR populations were retained. Subsequent Seurat analyses included data normalization, identification of the top 2000 highly variable genes, principal component analysis, dimensionality reduction, clustering using the top 30 principal components, UMAP visualization and differential gene expression testing with ‘FindAllMarkers’ using the Wilcoxon rank‐sum test (adjusted *p* value < 5×10^−6^; log_2_ fold change > 1.5).

##### Cell type annotation

Cell types were identified using established marker profiles,^33^ including mature neurons (*Syt1, Syn1, Rbfox3, Slc17a7, Gad1, Gad2*), immature neurons (*Dcx, Sox4, Sox11*), microglia (*Tmem119, Siglech*), oligodendrocytes (*Mog, Mbp, Mobp*), oligodendrocyte precursors (*Pdgfra*), astrocytes (*Gfap, Sox9*), and vascular leptomeningeal cells (*Dcn, Vtn*). Inhibitory populations were classified as CGE‐ or MGE‐derived based on *Vip* and *Reln, or Sst* and *Pvalb*, respectively. Mature glutamatergic neurons were assigned to Vglut2+ cells (*Slc17a6*) or to semilunar (*Ntng1, Slc17a7*) and pyramidal (*Slc30a3, Slc17a7*) types. These marker sets were then used by SCINA^73^ (https://github.com/jcao89757/SCINA) to generate automated annotations.

#### 4.4.2 Single‐cell methylation data analyses

Single‐cell DNA methylation profiles were examined using the dynamic browser at the Salk Institute Mouse Brain Atlas (https://mousebrain.salk.edu/dynamic_browser), which provides a high‐resolution transcriptomic and spatial atlas of the whole mouse brain including olfactory areas^47^ (OLF). Non‐CG methylation (mCH, where H = A, C, or T) was analysed as a cell‐type‐specific epigenetic signature, given its substantial variability among neuronal subtypes and negative correlation with gene expression, according to previous studies.^74^ Within OLF, mCH levels of *Slc17a7* were first used to identify glutamatergic neurons, followed by evaluation of *Reln*, *Rab3b*, *Ncam2* and *Abi3bp* in t‐SNE space to determine whether these markers could distinguish distinct glutamatergic neuronal subclusters.

#### 4.4.3 Single‐nuclei ATAC‐seq data analysis

##### Data files

The gene activity matrix, fragment file and corresponding meta tables were obtained from a dataset probing accessible chromatin in over 800 000 individual nuclei across 45 regions of the adult mouse brain, including olfactory areas.^34^ For an initial exploratory assessment of chromatin accessibility patterns in olfactory areas, the pre‐computed gene activity matrix was downloaded from the CellxGene portal^75^ and clustered using standard Seurat workflows to generate the exploratory visualization in Figure S1D. For each gene, counts within the gene body and an additional 2 kb upstream region were summed to compute the activity score. The fragment file was downloaded from NCBI GEO (GSE173650) and contains all unique chromatin fragments associated with each individual cell, offering a comprehensive view of chromatin accessibility. All formal downstream analyses were performed using an independent processing pipeline starting from the raw fragment files.

##### Fragment file pre‐processing

The snATAC‐seq data were processed using the Signac package (v1.12.0)^76^ with default parameters. The fragment file was loaded with the CreateFragmentObject function, and peak calling was performed using the CallPeaks function. To quantify fragment counts within each peak, the FeatureMatrix function was applied. Seurat objects were then generated from these data using the CreateChromatinAssay function. Each sample's Seurat object was subjected to QC, filtering cells based on metrics including nucleosome signal, TSS enrichment, fragment count within peaks, and the fraction of fragments in genomic blacklist regions. After QC filtering, cells were normalized and embedded in a low‐dimensional space using latent semantic indexing (LSI) through the Signac functions FindTopFeatures, RunTFIDF and RunSVD. Subsequently, graph‐based clustering and non‐linear dimensional reduction were performed using Seurat functions (RunUMAP, FindNeighbors and FindClusters).

##### Chromatin accessibility analyses

Chromatin accessibility profiles were computed as the averaged frequency of sequenced DNA fragments within specific genomic regions for different cell groups, using the CoveragePlot function in Signac. Gene activity scores were then calculated as an estimate of gene expression levels in snATAC‐seq data, where higher scores indicate greater chromatin accessibility around genes. Specifically, gene coordinates were extended by 2 kb upstream, and fragment counts within these regions were quantified using the FeatureMatrix function, with the entire process streamlined by the GeneActivity function. These scores were used for integrative analyses with snRNA‐seq data.

##### Cell type annotation

Canonical correlation analyses (CCA) were first performed to generate a shared dimensionality reduction of gene expression in the ‘reference’ snRNA‐seq data^33^ and gene activity in the ‘query’ snATAC‐seq data.^34^ With well‐annotated PN subtypes of snRNA‐seq data^33^ serving as the reference, snATAC‐seq subpopulations for PN subtypes were then annotated using Seurat's label‐transfer algorithm. After annotation, the results were validated by examining chromatin accessibility of PN subtype markers. Accessibility near *Ntng1* and *Slc30a3* distinguished SL and PYR neurons, while SL1 and SL2 were defined by *Reln* and *Rab3b*, and PYR1 and PYR2 by *Ncam2* and *Abi3bp*.

**Differentially accessible peak analysis** was conducted to identify chromatin regions with distinct accessibility across four PN subtypes. For each subtype, FindMarkers was applied to perform one‐vs‐all comparisons, using the Wilcoxon rank‐sum test with default parameters. Peaks with an adjusted *p* value less than 0.05 and an average log_2_ fold change greater than 0.25 were considered significantly differentially accessible (DAPs). To facilitate biological interpretation, the closest gene to each DAP was identified using the *ClosestFeature* function, providing potential regulatory candidates for further investigation.

##### Transcription factor binding motifs analysis

To identify cell‐type‐specific regulatory factors, overrepresented DNA motifs in DAPs between cell types were examined. The motif analyses began by retrieving motif data from JASPAR 2020,^77^ which was integrated into the snATAC‐seq dataset using the *AddMotifs* function in Signac. The *FindMotifs* function was then used to identify overrepresented motifs, performing a hypergeometric test internally to assess statistical significance. To investigate motif activity at the single‐cell level, chromVAR^78^ was used to compute a per‐cell motif accessibility matrix, representing the number of peaks containing each transcription factor motif across all cells. chromVAR then calculated deviation z‐scores for each motif, correcting for technical factors such as GC content bias, PCR amplification and variable Tn5 tagmentation. This workflow enables the identification of transcription factor binding motifs associated with variations in chromatin accessibility across different cell types.

#### 4.4.4 Spatial transcriptomics data analyses

**10x Spatial Visium data** was obtained from our previously published work,^53^ using 10x Genomics Visium platform to generate spatial transcriptomic data from the mouse forebrain (10x Spatial Visium, 55 µm spots; 20 µm thick brain slices; anterior‐posterior axes from –0.48 to +0.74 mm). Eight adjacent coronal sections were analysed, covering seven brain regions with a focus on the OLF regions. In total, 28 517 Visium spots were captured, 1214 of which were in the PIR region. These spots were manually mapped to three layer‐located regions (PIR layer1, 2, and 3). A gene‐spot count matrix with well‐annotated brain regions was used for all downstream analyses.

##### Pre‐processing

Seurat was used for gene‐spot matrices processing and downstream analyses. QC was performed for each gene‐spot matrix of brain slices, the spots with detected gene numbers under 200 were removed while the genes with fewer than 10 read counts or expressed in fewer than 2 spots were removed. Normalization across spots was performed with the *SCTransform* function. Highly variable genes (HVG) were selected using the *FindVariableFeatures* function in Seurat with the ‘vst’ setting for 5000 features; after scaling the feature data, principal component analysis (PCA) was performed with the first 30 principal components, and clustering was conducted using the Louvain algorithm at a resolution of 0.8. Spatial feature expression plots were generated with the ‘SpatialFeaturePlot’ function (Figure S2D).

##### PIR subregion registration

Spatial differential expression analysis identified several PIR‐enriched genes such as *Slc30a3*. This spatial enrichment was consistent with a lower‐resolution (100 µm spot) public whole‐brain spatial transcriptomic atlas^37^ generated using the 10x Spatial Visium platform (Figure S2A–C). Unsupervised clustering of the eight slices revealed two subregions within layer II, with high expression of *Ntng1* and *Slc30a3*, corresponding to SL and PYR types. Based on previous studies,^46^ these subregions are proposed to correspond to PIR layer IIa (superficial) and IIb (deep) (Figure S2D). Given that each Visium spot contains 2–6 cells, we estimated the proportion of each neuronal subtype using a deconvolution algorithm, using the established PN subtype labels defined in our prior analysis of public snRNA‐seq data^33^ as a reference (Figure S2F).

**Differential expression analysis of PIR2 subregion** was performed using the FindMarkers function in Seurat (Figure S2A). First, within our higher‐resolution (55 µm) 10x Visium dataset, gene expression in PIR2 was compared against the combined PIR1 and PIR3 subregions. Second, an analogous comparison (PIR2 vs. combined PIR1 and PIR3) was carried out in the whole‐brain Visium dataset with 100 µm resolution. For both analyses, genes were considered significantly enriched in PIR2 if they met the thresholds of an adjusted p value < .05 and a log_2_ fold change > .25.

##### Cluster‐based annotation of PIR layer II subregions

Unsupervised clustering was performed on 28 517 Visium spots from eight coronal brain slices, revealing two distinct clusters within PIR layer 2 (Figure S2B). One cluster exhibited high expression of *Ntng1*, a classic marker for SL cells, which are primarily located in the superficial layer of the PIR, commonly referred to as the 2a subregion.^46^ This cluster was annotated as the 2a subregion. The second cluster showed high expression of S*lc30a3*, a gene enriched in PYR cells in deeper layer II, which led to its annotation as the 2b subregion.

##### Spatial cell‐type deconvolution

The spot size of the Visium data is 55 µm, with each spot containing approximately 2–7 cells. Deconvolution analysis was performed using the spacexr package (v2.2.1)^79^ with default parameters to infer cellular composition in the spatial transcriptomics dataset. For this, snRNA‐seq data from Zeppilli 2025^33^ was used as a reference, providing well‐annotated PN subtypes. Both snRNA‐seq and 10CVisum data were loaded and subsequently converted into a reference object and a spatial RNA object, respectively. The *create.RCTD* and *run.RCTD* functions were then applied to perform the deconvolution and estimate cell type composition at each spatial location.

##### Spatial deconvolution in the whole‐brain wide Visium data

To characterize the broader spatial distribution of PN subtypes within the PIR, a whole‐brain wide spatial transcriptomics dataset (10x Visium, 100 µm spot resolution, AP from –2.6 mm to 2.245 mm) was re‐analysed.^37^ The dataset comprised 75 coronal sections spanning the anterior‐posterior axis of an adult mouse brain hemisphere, encompassing a total of 34 053 spots. Every spot was registered to the standard Allen Mouse Brain Atlas (CCFv3) with stereotaxic coordinates, including anterior‐posterior (AP), medial‐lateral (ML), and dorsal‐ventral (DV) axis value, as well as anatomical acronyms. The spot‐by‐gene count matrix and accompanying metadata were obtained. Spots annotated to the PIR were isolated for subsequent analysis. Cellular deconvolution was performed on these spots using spacexr package and a well‐annotated snRNA‐seq dataset from Zeppilli 2025 as a reference, enabling the estimation of proportions for PN subtypes and Vglut2+ neurons within each Visium spot. For visualization, spots were ordered according to their coordinate values along each independent anatomical axis,^18^ following the anterior (A) to posterior (P), dorsal (D) to ventral (V), and medial (M) to lateral (L) directions. The estimated cell‐type proportions were then plotted against these ordered positional values to delineate distribution trends.

##### Cellular spatial correlation analysis

To investigate the spatial relationships between PN subtypes and glial cells, a spatial correlation analysis was conducted using the deconvoluted proportions of Visium spots located within the PIR2 subregion of both the anterior and posterior piriform cortex (aPIR and pPIR) in the 55 µm resolution Spatial Visium dataset. The analysis was based on the matrix of cell‐type proportion estimates derived from prior spatial deconvolution, with rows representing spots and columns representing cell types. Pairwise correlations between the columns (i.e., across all Visium spots) were calculated for the estimated proportions of each major cell type, including PYR subtypes, inhibitory neurons (IN), Vglut2+ glutamatergic neurons, and glial cells, using Pearson's correlation coefficient. This analysis assessed whether the spatial distributions of any two cell types were positively or negatively associated across the tissue region.

##### RNA expression of PN Subtype‐specific markers in MERFISH and Stereo‐seq data

Single‐cell spatial transcriptomic data were obtained from two distinct sources: multiplexed error‐robust fluorescence in situ hybridization (MERFISH) data^35^ from the Salk Institute Mouse Brain Atlas (https://mousebrain.salk.edu/dynamic_browser), and Stereo‐seq data^36^ from the Mouse Spatial Transcriptomics Atlas (https://mouse.digital‐brain.cn/spatial‐omics/). MERFISH data were derived from the 35th coronal section covering the aPIR subregion, while Stereo‐seq data came from a coronal section at bregma +0.38 mm, also encompassing the aPIR. Normalized expression levels of *Reln*, *Rab3b*, *Ncam2* and Abi3bp were assessed to determine whether their spatial distributions were distinct. Gene counts in the MERFISH dataset were normalized by cell volume and log2‐transformed for visualization.

#### 4.4.5 Single‐neuron projection analyses via epi‐retro‐seq data

##### Pre‐processing

Epi‐retro‐seq, which combines retrograde labelling with single‐nucleus DNA methylation sequencing, was used to link the epigenomic properties of neurons to their long‐distance projection targets. epi‐retro‐seq data in ALLC format were obtained from GSE150170,^70^ including 1981 glutamatergic neurons from the aPIR and pPIR. The ALLC files were used to extract standardized non‐CG methylation (mCH) expression matrices and projection target metadata for each neuron, covering 10 targets: amygdala (AMY), hippocampal formation (HPF), anterior cingulate area (ACA), main olfactory bulb (MOB), prefrontal cortex (PFC), posterior parietal association area layerβ (PTLβ), striatum (STR), thalamus (TH), ventral tegmental area (VTA), and medulla (MY). The mCH‐normalized matrices and projection metadata were integrated to construct Seurat objects, followed by dimensionality reduction and clustering.

**Cell‐type annotation** was performed using SCINA with predefined subtype marker sets derived from the PIR snRNA‐seq reference dataset. Gene‐level mCH profiles were used as the input matrix for SCINA annotation. SL and PYR neurons were initially distinguished based on *Ntng1* and *Slc30a3* expression. SL1 and SL2 subclusters were defined by *Reln* and *Rab3b*, PYR1 and PYR2 were annotated by *Ncam2* and *Abi3bp*, and Vglut2+ cells identified by *Slc17a6*.

**Regional projection distribution statistics** were performed to quantify the output patterns of PIR glutamatergic neurons. Five source neuronal populations within the aPIR and pPIR were defined for analysis: the four molecularly defined PN subtypes (Reln+ SL, Rab3b+ SL, Abi3bp+ PYR, Ncam2+ PYR) and the broader Vglut2+ glutamatergic neuron population. For each defined source population, the proportion of its projections to a specific target region was calculated as the percentage obtained by dividing the number of neurons from that population projecting to the target region by the total number of neurons from the same population projecting to any of the ten target regions. The projection profiles for all five source populations from the aPIR and pPIR were compiled and visualized as a stacked area plot. In this plot, the *x*‐axis represents the different source neuronal populations. Each coloured layer corresponds to a target brain region. The thickness of a layer (its value on the *y*‐axis) at a given position along the x‐axis visually encodes the calculated proportion of projections from that specific source population innervating the corresponding target region. The combined height of all layers at any point on the *x*‐axis represents 100% of the total projections from that source population.

#### 4.4.6 Cross‐region comparisons of PN subtypes with broader cortical atlas

To contextualize our defined principal neuron (PN) subtypes in a broader cortical area, a snRNA‐seq dataset^33^(Zeppilli 2025) containing our defined PN subtypes was integrated with a mouse atlas across the isocortex and hippocampal formation^39^(Yao 2021). Both datasets were subsampled to 20 145 and 71 000 glutamatergic neurons, respectively. Data integration was performed using the Seurat CCA‐based integration pipeline with default parameters. The integrated dataset was visualized in two dimensions using UMAP. The resulting integrated clusters were analysed to quantify the co‐clustering of PIR‐derived PN subtypes with glutamatergic neurons from neocortical, transitional (EC, LEC, MEC, TPE‐ENT), and hippocampal formation (Sub, Sub‐ProS, PPP, RHP, DG, CA1‐3) areas. The degree of co‐clustering for PIR neurons was quantified as the fraction of PIR‐derived cells assigned to a given integrated cluster relative to the total number of PIR cells in the integrated dataset.

#### 4.4.7 Reference mapping of PN subtypes with the whole‐brain atlas

To harmonize PN subtype annotations with a standardized reference taxonomy, our snRNA‐seq dataset^33^ (Zeppilli et al.) was integrated with the mouse whole‐brain atlas^47^ (Yao et al.). A total of 38 526 PIR nuclei were extracted from the Yao atlas and annotated according to ‘supertype_label’ labels. Each subtype was randomly downsampled to a maximum of 1000 nuclei, while subtypes containing fewer nuclei were retained. After subsampling, 4921 and 8579 glutamatergic neurons from the Zeppilli and Yao datasets, respectively, were integrated using the Seurat CCA pipeline with default parameters. The correspondence between the four PN subtypes and atlas‐defined glutamatergic populations was quantified by calculating the proportion of PN subtype cells assigned to each integrated cluster, reflecting their co‐clustering with reference atlas populations.

### 4.5 Analyses of selective vulnerability of PNs in AD

#### 4.5.1 Association with AD Pathology

**Differential expression and functional enrichment analysis** were performed using the *FindMarkers* function in Seurat and the *enricher* function in clusterProfiler. First, within an snRNA‐seq dataset in which we defined four PN subtypes,^33^ normalized gene expression in Reln+ SLs was compared against Rab3b+ SLs. Genes were considered significantly enriched in Reln+ SLs if they met the thresholds of an adjusted *p* value < .05 and a log2 fold change > .25. Functional enrichment analysis was then performed on these genes using WikiPathways, with enriched pathways identified based on a significance threshold of adjusted *p* value < .05.

**A spatial transcriptomics dataset of AD mouse models with an Aβ plaque index** was used (from AppNL‐G‐F mice at ages 3, 6, 12 and 18 months). In this dataset,^43^ Aβ plaques were detected using 6E10 immunofluorescence staining. The Aβ index for each tissue domain (TD, defined for each spot with a diameter of 100 µm) was calculated as the standard deviation of Aβ fluorescence intensity across the pixels. The Aβ index was then log‐transformed for subsequent analysis, with higher values indicating a greater degree of plaque accumulation. Raw gene‐spot count matrices and metadata, including annotations for brain regions and Aβ plaque index, were downloaded. For further analysis, 750 TD regions from the ‘OLF’ area were retained.

##### Linking cell‐type deconvolution to spatial Aβ accumulation

The Visium data^43^ from AD mouse models, was used to investigate the spatial relationship between specific PN subtypes and local Aβ pathology. First, the cellular composition of each TD was inferred using the spacexr package, with snRNA‐seq data from Zeppilli 2025 serving as a reference for annotating PN subtypes. Next, to investigate the spatial association between a specific cell type and local Aβ pathology, Pearson correlation coefficients were calculated between the deconvoluted proportion of that cell type and the log‐transformed Aβ index across TDs. Prior to the correlation analysis, the distribution of the deconvoluted proportions was assessed for each cell type. A highly right‐skewed distribution was observed for PN subtypes across the entire PIR region, with a substantial fraction of TDs showing very low proportions (e.g., < 2%). This observation is consistent with the known concentration of PN subtypes in PIR layer 2. To focus the analysis on TDs where the cell type was sufficiently represented, a threshold was applied, including only those TDs where the cell‐type deconvolution proportion exceeded 2% in the correlation calculation.

#### 4.5.2 Spatial differential expression and cellular correlation

##### Spatial differential expression analysis

In this analysis,^43^ differential expression between Reln+ SL‐high expressing tissue domains (TDs) and the remaining TDs was investigated. To define the Reln+ SL‐enriched group, the top 30% of TDs with the highest deconvoluted proportions of Reln+ SL were selected, while the remaining 70% were used as the comparison group. Differentially expressed genes (DEGs) were identified between these two groups, resulting in 65 significant genes. KEGG and Wiki pathway enrichment analyses were subsequently performed on these DEGs using the clusterProfiler package, following the same method as described previously.

##### Spatial cellular correlation

To examine the spatial relationships between PN subtypes and glial cells in both wild‐type and AD mouse models, a spatial correlation analysis was conducted using the deconvoluted proportions of TD spots located within the entire PIR region of the 100 µm resolution Spatial Visium dataset. The analysis was performed according to the methods outlined earlier.

#### 4.5.3 Cell‐cell communication inference

**High‐resolution Stereo‐seq data of AD mouse models** from AppNL‐G‐F KI and WT mice at 3 and 18 months of age were analysed, comprising 122 931 single cells of olfactory regions from seven samples (four AD and three WT).^40^ Upon obtaining the expression matrix, clustering, dimensionality reduction, and annotation were performed in Seurat pipeline. Cell types were identified based on established marker gene profiles. Mature neurons were classified using markers such as *Syt1, Syn1, Rbfox3, Slc17a7, Gad1* and *Gad2*. Microglia, oligodendrocytes, astrocytes, and vascular leptomeningeal cells were identified by markers *Tmem119, Siglech, Mog, Mbp, Mobp, Gfap* and *Sox9*, and *Dcn* and *Vtn*, respectively. Inhibitory populations were further classified into CGE‐ or MGE‐derived groups based on expression of *Vip* and *Cck*, or *Sst* and *Pvalb*. Mature glutamatergic neurons were assigned to Reln+ SL (marked by *Ntng1, Slc17a7, Reln*) and Ncam2+ PYR (marked by *Slc30a3, Slc17a7, Ncam2*), with additional unclassified neuronal types (e.g., refer to Neu1, Neu2, *Slc17a7*) identified.

**Cell–cell communication** analyses were then performed using the R package CellChat with default parameters. The CellChatDB mouse was used for analyses. To enable comparative analyses, the gene expression matrices from all six sample groups were first normalized and integrated together using the *NormalizeData* and *FindIntegrationAnchors* functions in Seurat, ensuring a common gene expression space. Subsequently, the integrated Data were split by group for individual CellChat analyses. Statistically significant secreted signalling interactions were identified within each age‐by‐disease group. These interactions were then filtered to retain only those that were significant under AD conditions but non‐significant in the age‐matched WT mice. To ensure robustness, this AD‐specific pattern was further required to be reproducible across independent age groups. Through this sequential filtering, the GRN and SPP1 signalling pathways were ultimately identified as communication events that were consistently and specifically altered in AD.

#### 4.5.4 Cross‐regional expression analyses

The snRNA‐seq dataset from Zeppilli,^33^ which includes annotations of PN subtypes within the PIR 2a region, was analysed together with the single‐cell cortical atlas from Yao,^39^ both of which were derived from WT mice. The latter dataset provides detailed annotations for various brain regions, including the hippocampus (CA1, CA3) and EC subregions (EC layer II and III), along with corresponding cell type labels. Our analysis focused on the relative expression levels of *Sort1* and *Gpc5* across these regions. Based on prior studies,^3^ excitatory neurons in CA1 and EC layer II were categorized as ‘vulnerable neurons’, while those in CA3 and EC layer III were classified as ‘resilient neurons’. Consistent with our previous analysis, Reln+ SLs were classified as ‘vulnerable neurons’, whereas Rab3b+ SLs were labelled as ‘resilient neurons’.

#### 4.5.5 Gene expression changes during normal aging

Gene expression data^41^ for *Sort1* and *Gpc5* were obtained from the publicly available dataset at https://alz.princeton.edu/. This dataset used the bacTRAP (Bacterial Artificial Chromosome‐Translating Ribosome Affinity Purification) technology to generate molecular profiles of seven neuronal types that are highly vulnerable or relatively resistant to Alzheimer's disease. It includes single‐nucleus transcriptomes of glutamatergic neurons across the EC and hippocampal regions at three age points (5, 12 and 24 months). Specifically, the bacTRAP gene expression (RPKM) values and their standard deviations were downloaded. This enabled us to examine changes in the average gene expression of both genes in wild‐type mice during normal aging, with a focus on the transitions from 5 to 12 months and from 12 to 24 months.

#### 4.5.6 AD risk association analysis

The analysis incorporated two distinct risk scores to assess the association of target genes with late‐onset Alzheimer's disease (AD): the Genetic Risk Score (GRS) and the Multi‐omics Risk Score (MRS). The GRS summarizes genetic evidence from various studies, including genome‐wide association studies (GWAS), proxy GWAS (GWAX) and quantitative trait loci (QTL) research. In contrast, the MRS combines both transcriptomic and proteomic evidence to provide a more comprehensive view of gene‐disease associations at the molecular level. Detailed descriptions of the data sources and methodologies used to generate these scores are available in https://alz‐journals.onlinelibrary.wiley.com/doi/10.1002/trc2.12461. Specifically, both genetic and multi‐omics risk scores for approximately 24 395 genes were obtained from https://agora.adknowledgeportal.org/. The GRS distribution spanned from 0 to 3, while the MRS scores ranged from 0 to 2. The GRS followed a normal distribution, allowing the use of standard *t*‐tests to assess whether individual genes showed significantly elevated risk scores. In contrast, the MRS followed a negative binomial distribution, and thus Monte Carlo simulations were used to assess the disease risk levels of these genes based on the overall distribution of scores.

#### 4.5.7 Gene expression associated with Braak staging

##### Cross‐regional expression in human AD samples

To determine whether the gene expression patterns observed in mouse models are also reflected in human AD samples, we used a multi‐regional human AD dataset.^9^ This snRNA‐seq dataset includes 1.3 million cells from 283 post‐mortem human brain samples across six brain regions, representing 48 individuals at various stages of AD progression. The data were accessed via the online platform at http://compbio2.mit.edu/ad_multiregion/, where violin plots of pseudo‐bulk gene expression for *Sort1* and *Gpc5* were retrieved, averaged across cell types in each unique combination of cell type and sample. For each author‐annotated cell type, brain region were manually assigned based on the corresponding brain region‐cell type mapping diagram from the original study.^9^ Consistent with previous studies, ‘CA1 pyramidal cells’ from the hippocampus, ‘Exc L5 AGBL1 GPC5’, and ‘Exc L2‐LEC RELN+GPC5+’, ‘Exc L3 RELN+COL5A2+’, ‘Exc L2/3 TOX3+TTC6+’ in the EC were annotated as ‘vulnerable neurons’, while ‘CA2 and CA3 pyramidal cells’ and ‘DG granule cells’ were annotated as ‘resilient neurons’.

##### Gene expression grouped by Braak staging

As each sample in this snRNA‐seq dataset is associated with a Braak stage, the expression data were further stratified based on these stages. The expression of *Sort1* and *Gpc5* was then visualized across these stages on the platform http://compbio2.mit.edu/ad_multiregion/ by using the ‘Braaksc’ attribute, allowing us to examine the distribution of gene expression within each Braak stage through violin plots

### 4.6 Analysis of molecular programs distinguishing LEC and MEC

#### 4.6.1 Analysis of vulnerability‐associated gene expression

A published single‐nucleus RNA‐seq dataset from whole brains of WT and 5XFAD mice was re‐analysed.^60^ Raw count matrices and cell‐type annotations (predicted.id) were obtained from GSE331294. L2 ENTl, L2 ENTm, and L3 ENT excitatory neurons were subsetted and processed using the Seurat workflow. Cell identities were validated based on canonical marker expression (Figure S5C), including *Bcl11b* for L2 ENTl and *Dcc* for L2 ENTm neurons according to previous studies.^80^ Differential expression analysis was performed using the Wilcoxon rank‐sum test with FDR correction. Expression of *Reln*, *Gpc5* and *Sort1* were subsequently compared across neuronal populations.

#### 4.6.2 Weighted gene co‐expression network analysis (WGCNA)

High‐dimensional weighted gene co‐expression network^81^ (hdWGCNA) analysis was performed independently within each neuronal population and condition (L2 ENTl, L2 ENTm and L3 ENT from WT and AD mice). Genes detected in at least 5% of nuclei were selected, and nuclei were aggregated into metacells using the hdWGCNA *MetacellsByGroups* function based on the UMAP nearest‐neighbour graph (k = 5). Co‐expression networks were constructed from metacell expression profiles using soft‐thresholding parameters selected based on scale‐free topology criteria, followed by module identification using the hdWGCNA workflow. Modules detected exclusively in the AD ENTl network, but not in corresponding ENTm or L3 ENT networks, were selected for downstream analyses.

#### 4.6.3 Expression changes in the 52‐gene module in AD versus WT

To assess the coordinated regulation of the 52‐gene module, normalized average expression values were computed for L2 ENTl, L2 ENTm and L3 ENT neurons from the same snRNA‐seq dataset^60^ under both WT and AD conditions. Within each cell type, individual genes were classified as up‐ or down‐regulated based on their calculated fold changes between AD and WT. Directional consistency of the module was then evaluated using a binomial test (*binom.test* function in R), which tested whether the observed proportion of AD‐upregulated genes significantly exceeded the null expectation of 0.5.

#### 4.6.4 Validation of the ENTl‐associated gene module in Yao (2021)

To evaluate whether the identified molecular program represented an intrinsic molecular feature of ENTl neurons, an independent mouse brain snRNA‐seq atlas of normal mice from Yao^39^ was analysed. L2 ENTl, L2 ENTm and L3 ENT neurons were extracted, and dimensionality reduction and clustering analyses were performed in Seurat using the module genes as input features. Specifically, data were normalized, and the module genes were used as the feature set for principal component analysis (PCA) with the first five dimensions were retained (dims = 1:5). Neighbour graphs were constructed and clusters were identified at a resolution of 0.5, followed by *t*‐SNE visualization. The resulting clustering was compared with that obtained using randomly selected gene sets of matched size (dims = 1:5) and the top 2000 highly variable genes (dims = 1:10).

#### 4.6.5 Functional enrichment analysis of gene module

Functional enrichment analysis was performed on the genes of the ENTl‐associated molecular program using the clusterProfiler package with mouse genome annotation (org.Mm.eg.db). Over‐representation analyses were conducted for Gene Ontology (GO) terms, UniProt keywords and cellular component annotations. Significantly enriched terms were identified based on FDR‐adjusted *p* value below .05.

#### 4.6.6 i‐cisTarget analysis of cis‐regulatory signatures

To identify enriched cis‐regulatory signatures associated with the Sort1‐containing gene module, i‐cisTarget analysis^82^ (https://gbiomed.kuleuven.be/apps/lcb/i‐cisTarget/) was performed using the 52‐gene module as input. Mouse genes were first converted to their human orthologs, yielding 49 homologous genes, which were then submitted as a gene list. The analysis was conducted against the *Homo sapiens* (hg19) reference genome (RefSeq release 45) using database version 6.0. A full analysis was performed employing the whole motif collection. Enrichment was assessed against curated regulatory feature databases, including transcription factor binding motifs, transcription factor binding sites, chromatin accessibility‐associated regions (DHS/FAIRE), and histone modification profiles. Regulatory features with normalized enrichment scores (NES) > 3.0 were considered significantly enriched.

For each significantly enriched regulatory feature (NES > 3.0), the top‐ranked cis‐regulatory regions and their associated genes were extracted from the i‐cisTarget output. These regions represent those with the highest signal strength for that specific feature in the global ranking. The frequency of each of the 52 module genes among these top‐ranked regions was then calculated across all enriched features, allowing the identification of genes repeatedly associated with multiple high‐confidence regulatory signatures.

### 4.7 Statistical analyses and visualization

#### 4.7.1 Statistical methods

Differential gene expression analyses were performed using the Wilcoxon rank‐sum test. For the Alzheimer’ s disease (AD) risk association analyses, the overall genetic risk scores of approximately 20 000 genes were found to follow a normal distribution and were assessed using a one‐sample *t*‐test against the null hypothesis mean. In contrast, multi‐omics risk scores followed a negative binomial distribution, and their significance was evaluated using Monte Carlo simulations. Multiple testing was corrected using the false discovery rate (FDR) method. A significance threshold of .05 was applied, with significant results marked by a single asterisk. Statistical significance is indicated as *p < .05, **p < .01, ***p < .001.

#### 4.7.2 Software and Packages

Single‐cell and spatial transcriptomics data were processed and analysed using Seurat and Scanpy^83^ (v1.9.3). Single‐cell chromatin accessibility data (snATAC‐seq) was analysed with Signac (v1.12.0). All statistical analyses and data visualizations were performed in R (v4.2.2) or Python (3.10.11). Figures were generated using ggplot2 (v3.4.1) with colour schemes from ggsci (v2.9) and statistical annotations from ggpubr (v0.6.0). Three‐dimensional representations of the LEC and MEC were generated through the SMDB.^84^

## AUTHOR CONTRIBUTIONS

Guoqing Zhang, Liyun Yuan and Yaya Zhao conceived of the project, supervised the study and revised the manuscript. Jiayue Meng analysed the single‐cell multi‐omics data and wrote the manuscript. Miaomiao Zhang performed the immunostaining experiment and wrote the manuscript. Yujie Chen, Nianhao Cheng and Mengyao Han performed the investigation. Ruifang Cao provided the methodology. Qinwen He performed the visualization.

## CONFLICT OF INTEREST STATEMENT

The authors declare no conflicts of interest.

## Supporting information




**Figure S1**. Molecular validation of the four PIR PN subtypes.
**A**: Immunofluorescence staining for the PYR2 marker NCAM2 (left) and glutamatergic neuronal marker (VGLUT1/SlC17A7) in the human PIR (data from the Human Protein Atlas). **B**: RNA in situ hybridization images from Allen ISH Atlas validating the laminar expression of PN subtype markers (eg: *Reln, Rab3b, Ncam2*). **C**: Single‐nucleus DNA methylome (snmC‐seq2) profiles^47^ showing differential methylation levels (5mC in CH contexts, mCH) at the genomic loci of subtype‐defining marker genes. **D**: Gene activity scores derived from scATAC‐seq data^34^ with 48 615 PIR PNs, supporting subtype‐specific chromatin accessibility at subtype‐specific marker genes. **E**: Normalized RNA expression of subtype markers (*Reln, Rab3b, Abi3bp, Ncam2*) and key subtype‐associated transcription factors (e.g., *Rorb, Nfib*) in snRNA‐seq data.^33^

**Figure S2**. Regional distribution and molecular signatures of the four PN subtypes in the PIR.
**A**: Workflow for analysing spatial transcriptomics data. **B**: Validation of regionally enriched genes. Scatter plot comparing the expression fold‐change of PIR‐enriched genes identified in this study against a whole‐brain spatial transcriptomic atlas.^37^ **C**: Sublaminar organization of PIR layer II. Unsupervised clustering of spatial transcriptomics data resolves two clusters within PIR layer II, overlaid with the expression of SL and PYR marker genes. **D**: Spatial distribution of deconvoluted proportions for four PN subtypes across the PIR layer II. **E**: High‐resolution spatial expression of subtype markers. Expression patterns of key markers shown using 10x Visium^53^ (top) and single‐cell resolution Stereo‐seq^36^ (bottom) datasets. **F**: Methylation levels (mCH) of subtype markers and key transcription factors in an epi‐retro‐seq dataset.^70^

**Figure S3**. Molecular characterization and Aβ‐associated features of the Reln+ SL subtype.
**A**: Detailed composition of integrated cluster 0 from Figure 3A, showing the diverse cortical subregions contributing to the isocortical layer II/III intratelencephalic (IT) neuron population that co‐clusters with PIR Ncam2+ PYR neurons. **B**: Expression of canonical regional marker genes (*Dcc* for EC layer II, *Trps1* for EC layer III, *Cux2* for isocortex layer II) across the four PN subtypes in snRNA‐seq data. **C**: Representative multiplex immunofluorescence images showing Reelin expression and Aβ accumulation in the piriform cortex of AD model mice at 20 and 50 weeks of age. Reelin (red) was co‐stained with Aβ (green), with nuclei counterstained using DAPI (blue). Scale bar, 20 µm (applies to all fields). **D**: Representative multiplex immunofluorescence images showing Aβ accumulation and GFAP expression in the piriform cortex of AD model mice. GFAP (yellow) was used to visualize astrocytic responses associated with Aβ pathology. Scale bar, 50 µm (applies to all fields). **E**: RNA expression of cell type markers in a Stereo‐seq dataset.^40^

**Figure S4**. Vulnerability‐associated molecular signatures across mouse and human neuronal populations.
**A**: Expression of *Sort1* and *Gpc5* in glutamatergic neurons across three brain regions using published snRNA‐seq datasets. Vulnerable‐resilient pairs included hippocampal CA1 versus CA3, L2 IT ENT versus L3 IT ENT, and PIR Reln+ SL versus Rab3b+ SL neurons. **B**: Expression of vulnerability‐associated genes *GPC5* and *SORT1* in vulnerable and non‐vulnerable neuronal populations from normal human brains. Violin plots show pseudobulk expression across excitatory neuronal populations in the EC and hippocampus. Populations corresponding to vulnerable neurons are highlighted in blue.
Expression values were calculated as the average expression for a region within a donor.
**C**: Expression of region‐associated marker genes (*Reln*, *Dcc*, and *Bcl11b*) across mouse ENT excitatory neurons. **D**: Composition of integrated cluster 2 and remaining clusters based on cell‐type annotation, showing enrichment of L2 IT ENTl neurons. **E**: Comparison of feature sets for neuronal population separation using the independent reference dataset^39^ (Yao 2021). t‐SNE visualization of ENT excitatory neurons based on either a randomly selected set of 52 genes (top) or the top 2000 highly variable genes (bottom) as input features. Cells are coloured according to their original cell‐type annotations.

**Figure S5**. Integration and annotation harmonization across multi‐omics datasets.
**A‐C**: Integration of multiple PIR samples from a snRNA‐seq dataset.^33^ UMAP visualization of integrated PIR PNs coloured by sample identity (A), anatomical region (B), and cell‐type annotation (C), demonstrating reduced sample‐associated variation while maintaining biological cell‐type structure. **D–F**: Integration of PIR PNs onto a published mouse isocortical atlas.^39^ UMAP visualization of the integrated PIR snRNA‐seq dataset coloured by cortical brain region annotations (D), PIR reference dataset annotations (E), and whole‐brain reference atlas annotations (F). **G–I**: Cross‐modality integration and annotation transfer between snRNA‐seq and scATAC‐seq datasets. UMAP visualization of co‐embedded snRNA‐seq and scATAC‐seq datasets (G), and individual scATAC‐seq (H) and snRNA‐seq reference (I) datasets coloured by transferred PN subtype annotations. **J**: Reference mapping of PIR PNs across established PIR taxonomies. UMAP visualization of PIR neurons from Zeppilli 2025^33^ (middle), Yao 2023^47^ (bottom), and their co‐embedded representation after integration (top). **K**: Correspondence between integrated clusters and original cell‐type annotations. Heatmap showing the proportion of cells from each annotated population assigned to each integrated cluster.

**Table S1**. Detailed metadata of the public multi‐omics datasets used in this study.
This table summarizes all public datasets used in this study, encompassing single‐cell transcriptomic, epigenomic, spatial transcriptomic and joint projection‐methylome profiles. For each dataset, we list its specific purpose in our analysis, experimental platform, biological sample characteristics (species, genotype, age, sex, brain region), sample, cell or spot counts, data source, associated pathology metrics (where applicable) and primary publication. Brain region abbreviations are defined in the table footnote. The data type ‘single‐cell joint projection and methylome profiling data’ refers specifically to epi‐retro‐seq, a method that links single‐neuron projection targets to their methylome. Pathology annotations: “continuous beta‐amyloid plaques score” indicates a quantitative measure of plaque density, and ‘pathologic Braak stages’ refers to the post‐mortem neurofibrillary tangle staging for human samples. For spatial datasets, the “single‐cell number” column denotes the total number of spatial spots (or cells for MERFISH) profiled.
**Table S2**. Summary of experimental mice used for immunofluorescence analysis.
This table details the experimental animals for immunofluorescence staining. All tissues were obtained from male mice on a C57BL/6J genetic background, comprising an AD model (5XFAD) and WT mice. Tissues were collected at two critical age points: young adulthood (20 weeks) and middle age (50 weeks).

## Data Availability

All single‐cell and spatial omics data used in this study are publicly available. Specifically, data for defining and validating neuronal subtypes (GSE239477, GSE132489, and GSE173650), analysing their spatial distribution and projections (OEP004212, GSE147747, GSE150170, and CNP0003837), and cross‐brain region comparisons (GSE185862 and GSE246717) can be accessed from the respective repositories. Gene expression dynamics during normal aging were obtained from https://alz.princeton.edu/(GSE151460). Data related to Alzheimer's disease susceptibility, including spatial transcriptomics of disease models (GSE152506, GSE263789) and single‐cell nuclear RNA sequencing (GSE212606, GSE296091 and GSE331294), as well as multi‐region human brain single‐cell data from patients (syn52293442, available via http://compbio2.mit.edu/ad_multiregion/), are also publicly accessible. Detailed sources and identifiers for all datasets are provided in the ‘Materials and Methods’ section.
