# Supplementary material for: Integrative analysis of single‐cell multi‐omics reveals heterogeneity in piriform cortex principal neurons and their selective vulnerability in Alzheimer's disease
Source: Clin Transl Med. 2026 Sep 11;16(9):e70781. doi: 10.1002/ctm2.70781 (PMC13570217; doi:10.1002/ctm2.70781)
Supplement: Supplementary file 1 [file CTM2-16-e70781-s001.pdf]

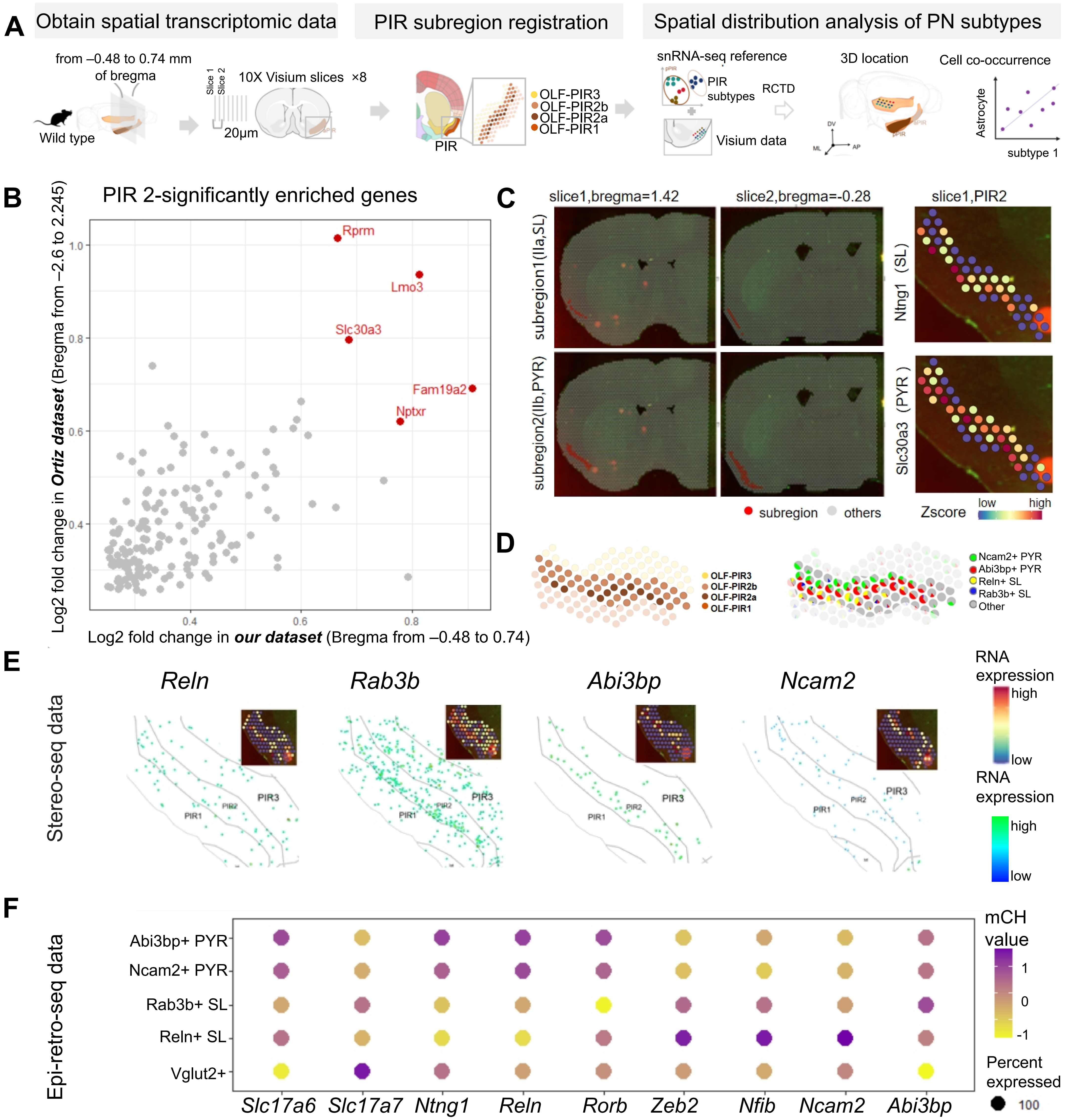

Figure S2. Regional distribution and molecular signatures of the four PN subtypes in the PIR.
A: Workflow for analyzing spatial transcriptomics data. **B:** Validation of regionally enriched genes. Scatter plot comparing the expression fold-change of PIR-enriched genes identified in this study against a whole-brain spatial transcriptomic atlas[36] . **C:** Sublaminar organization of PIR layer II. Unsupervised clustering of spatial transcriptomics data resolves two clusters within PIR layer II, overlaid with the expression of SL and PYR marker genes. **D:** Spatial distribution of deconvoluted proportions for four PN subtypes across the PIR layer II. **E:** High-resolution spatial expression of subtype markers. Expression patterns of key markers shown using 10x Visium[46] (top) and single-cell resolution Stereo-seq[35] (bottom) datasets. **F:** Methylation levels (mCH) of subtype markers and key transcription factors in an Epi-Entro-seq dataset[75].
