# Supplementary material for: Integrative analysis of single‐cell multi‐omics reveals heterogeneity in piriform cortex principal neurons and their selective vulnerability in Alzheimer's disease
Source: Clin Transl Med. 2026 Sep 11;16(9):e70781. doi: 10.1002/ctm2.70781 (PMC13570217; doi:10.1002/ctm2.70781)
Supplement: Supplementary file 2 [file CTM2-16-e70781-s002.pdf]

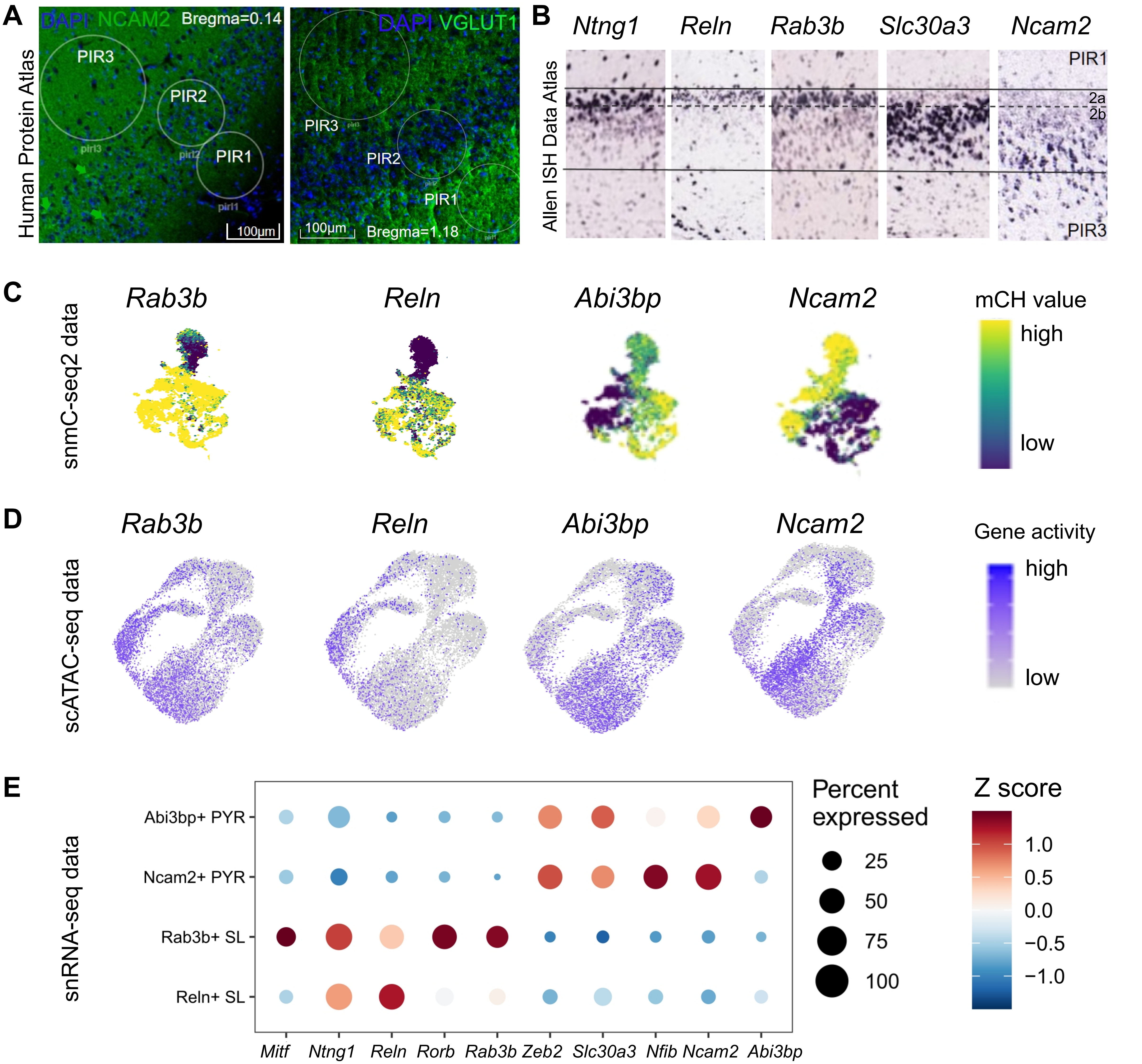

Figure S1. Molecular validation of the four PIR PN subtypes.

A: Immunofluorescence staining for the PYR2 marker NCAM2 (left) and glutamatergic neuronal marker (VGLUT1/SlC17A7) in the human PIR (data from the Human Protein Atlas). **B:** RNA in situ hybridization images from Allen ISH Atlas validating the laminar expression of PN subtype markers (eg: *Reln, Rab3b, Ncam2*). **C:** Single-nucleus DNA methylome (snmC-seq2) profiles[68] showing differential methylation levels (5mC in CH contexts, mCH) at the genomic loci of subtype-defining marker genes. **D:** Gene activity scores derived from scATAC-seq data[33] with 48,615 PIR PNs, supporting subtype-specific chromatin accessibility at subtype-specific marker genes. **E:** Normalized RNA expression of subtype markers (*Reln, Rab3b, Abi3bp, Ncam2*) and key subtype-associated transcription factors (e.g., *Rorb, Nfib*) in snRNA-seq data[32].
