# Supplementary material for: Integrative analysis of single‐cell multi‐omics reveals heterogeneity in piriform cortex principal neurons and their selective vulnerability in Alzheimer's disease
Source: Clin Transl Med. 2026 Sep 11;16(9):e70781. doi: 10.1002/ctm2.70781 (PMC13570217; doi:10.1002/ctm2.70781)
Supplement: Supplementary file 3 [file CTM2-16-e70781-s003.pdf]

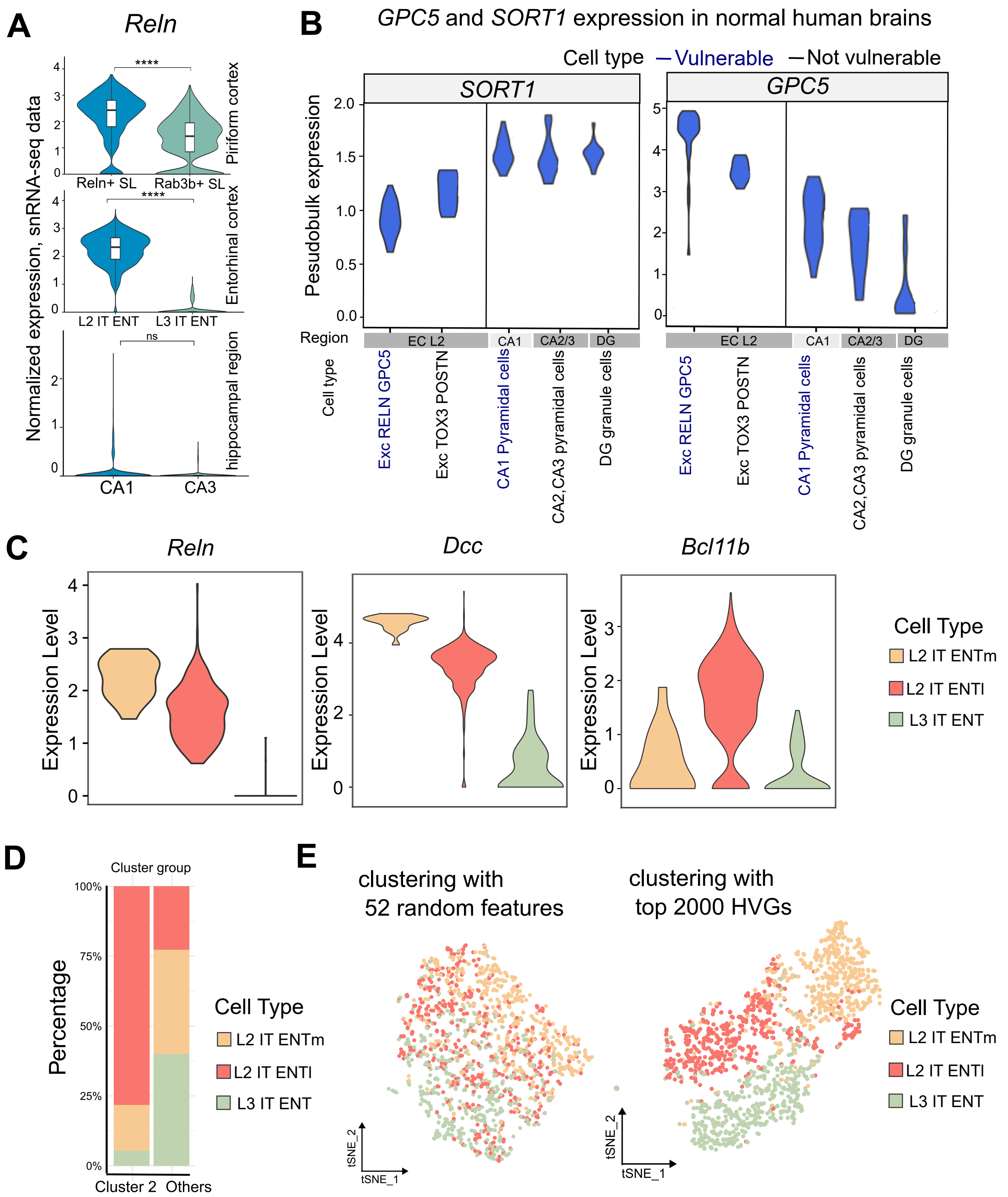

Figure S4. Vulnerability-associated molecular signatures across mouse and human neuronal populations

A: Expression of *Sort1* and *Gpc5* in glutamatergic neurons across three brain regions using published snRNA-seq datasets. Vulnerable-resilient pairs included hippocampal CA1 versus CA3, L2 IT ENT versus L3 IT ENT, and PIR Reln+ SL versus Rab3b+ SL neurons. **B:** Expression of vulnerability-associated genes *GPC5* and *SORT1* in vulnerable and non-vulnerable neuronal populations from normal human brains. Violin plots show pseudobulk expression across excitatory neuronal populations in the EC and hippocampus. Populations corresponding to vulnerable neurons are highlighted in blue. Expression values were calculated as the average expression for a region within a donor. **C:** Expression of region-associated marker genes (*Reln*, *Dcc*, and *Bcl11b*) across mouse ENT excitatory neurons. **D:** Composition of integrated cluster 2 and remaining clusters based on cell-type annotation, showing enrichment of L2 IT ENTl neurons. **E:** Comparison of feature sets for neuronal population separation using the independent reference dataset[38] (Yao 2021). t-SNE visualization of ENT excitatory neurons based on either a randomly selected set of 52 genes (top) or the top 2,000 highly variable genes (bottom) as input features. Cells are colored according to their original cell-type annotations.
