# Supplementary material for: Integrative analysis of single‐cell multi‐omics reveals heterogeneity in piriform cortex principal neurons and their selective vulnerability in Alzheimer's disease
Source: Clin Transl Med. 2026 Sep 11;16(9):e70781. doi: 10.1002/ctm2.70781 (PMC13570217; doi:10.1002/ctm2.70781)
Supplement: Supplementary file 4 [file CTM2-16-e70781-s004.pdf]

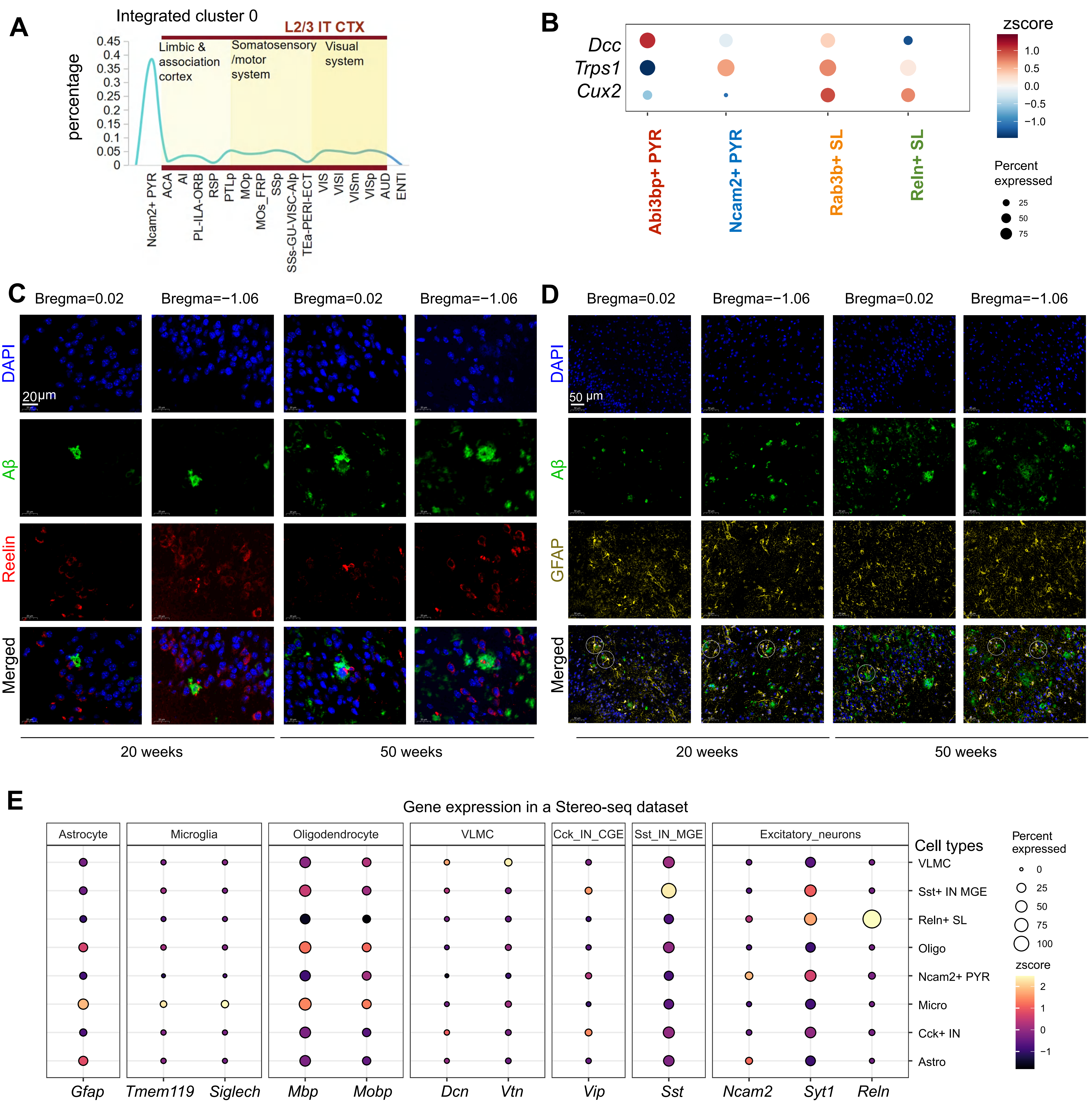

Figure S3. Molecular characterization and Aβ-associated features of the Reln+ SL subtype.

A: Detailed composition of integrated cluster 0 from Figure 3A, showing the diverse cortical subregions contributing to the isocortical layer II/III intratelencephalic (IT) neuron population that co-clusters with PIR Ncam2+ PYR neurons. **B:** Expression of canonical regional marker genes (*Dcc* for EC layer II, *Trps1* for EC layer III, *Cux2* for isocortex layer II) across the four PN subtypes in snRNA-seq data. **C:** Representative multiplex immunofluorescence images showing Reelin expression and Aβ accumulation in the piriform cortex of AD model mice at 20 and 50 weeks of age. Reelin (red) was co-stained with Aβ (green), with nuclei counterstained using DAPI (blue). Scale bar, 20 μm (applies to all fields). **D:** Representative multiplex immunofluorescence images showing Aβ accumulation and GFAP expression in the piriform cortex of AD model mice. GFAP (yellow) was used to visualize astrocytic responses associated with Aβ pathology. Scale bar, 50 μm (applies to all fields). **E:** RNA expression of cell type markers in a Stereo-seq dataset[39].
