# Supplementary material for: Integrative analysis of single‐cell multi‐omics reveals heterogeneity in piriform cortex principal neurons and their selective vulnerability in Alzheimer's disease
Source: Clin Transl Med. 2026 Sep 11;16(9):e70781. doi: 10.1002/ctm2.70781 (PMC13570217; doi:10.1002/ctm2.70781)
Supplement: Supplementary file 5 [file CTM2-16-e70781-s005.pdf]

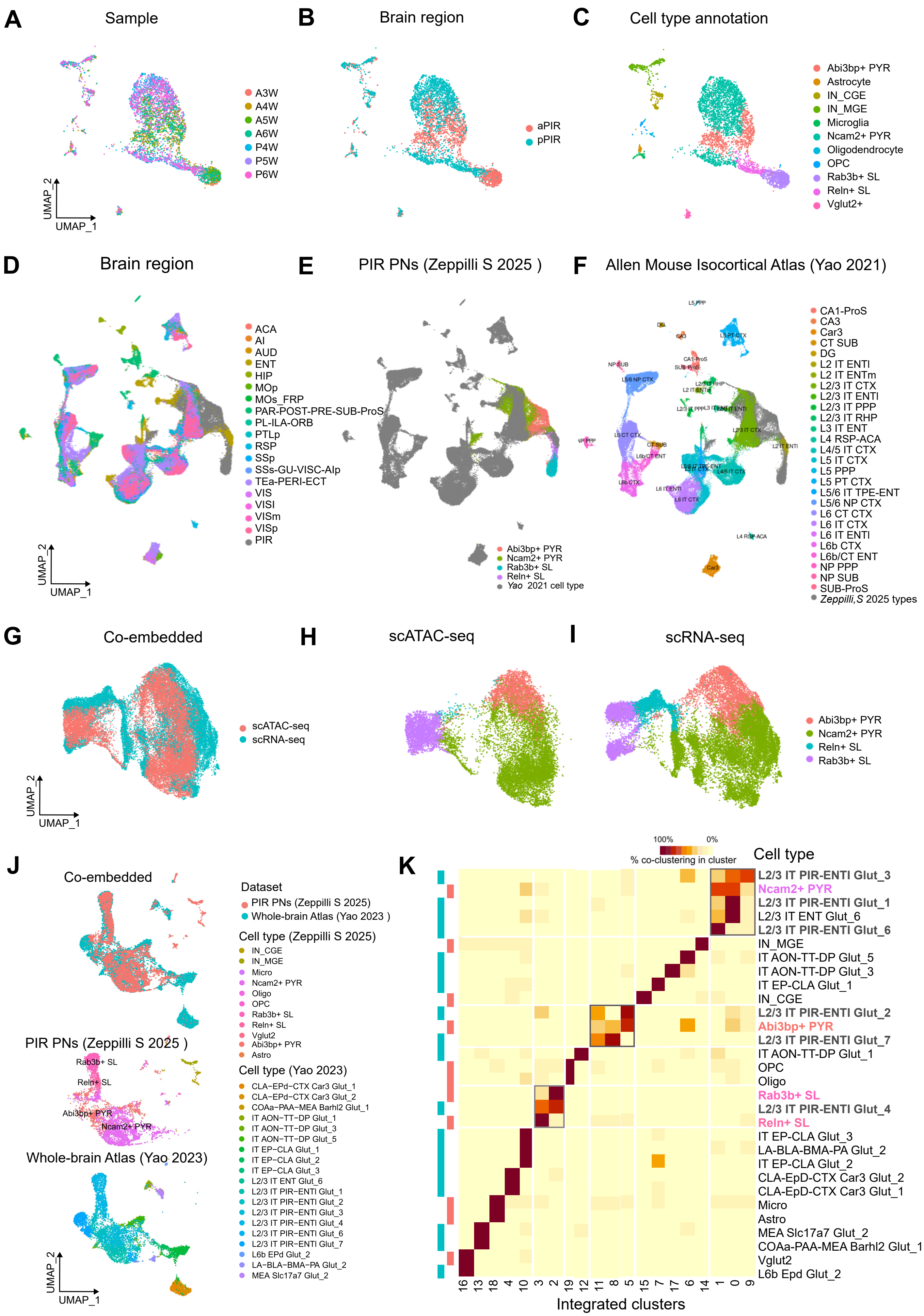

A
Sample
A3W
A4W
A5W
A6W
P4W
P5W
P6W
UMAP_2
UMAP_1
B
Brain region
aPIR
pPIR
C
Cell type annotation
Abi3bp+ PYR
Astrocyte
IN_CGE
IN_MGE
Microglia
Ncam2+ PYR
Oligodendrocyte
OPC
Rab3b+ SL
Reln+ SL
Vglut2+
D
Brain region
ACA
AI
AUD
ENT
HIP
MOp
MOs_FRP
PAR-POST-PRE-SUB-ProS
PL-ILA-ORB
PTLp
RSP
SSp
SSs-GU-VISC-AIp
TEa-PERI-ECT
VIS
VISl
VISm
VISp
PIR
UMAP_2
UMAP_1
E
PIR PNs (Zeppilli S 2025)
Abi3bp+ PYR
Ncam2+ PYR
Rab3b+ SL
Reln+ SL
Yao 2021 cell type
F
Allen Mouse Isocortical Atlas (Yao 2021)
CA1-ProS
CA3
Car3
CT SUB
DG
L2 IT ENTl
L2 IT ENTm
L2/3 IT CTX
L2/3 IT ENTl
L2/3 IT PPP
L2/3 IT RHP
L3 IT ENT
L4 RSP-ACA
L4/5 IT CTX
L5 IT CTX
L5 PPP
L5 PT CTX
L5/6 IT TPE-ENT
L5/6 NP CTX
L6 CT CTX
L6 IT CTX
L6 IT ENTl
L6b CTX
L6b/CT ENT
NP PPP
NP SUB
SUB-ProS
Zeppilli,S 2025 types
G
Co-embedded
scATAC-seq
scRNA-seq
UMAP_2
UMAP_1
H
scATAC-seq
I
scRNA-seq
Abi3bp+ PYR
Ncam2+ PYR
Reln+ SL
Rab3b+ SL
J
Co-embedded
PIR PNs (Zeppilli S 2025)
Rab3b+ SL
Reln+ SL
Abi3bp+ PYR
Ncam2+ PYR
Whole-brain Atlas (Yao 2023)
UMAP_2
UMAP_1
Dataset
PIR PNs (Zeppilli S 2025)
Whole-brain Atlas (Yao 2023)
Cell type (Zeppilli S 2025)
IN_CGE
IN_MGE
Micro
Ncam2+ PYR
Oligo
OPC
Rab3b+ SL
Reln+ SL
Vglut2
Abi3bp+ PYR
Astro
Cell type (Yao 2023)
CLA-EPd-CTX Car3 Glut_1
CLA-EPd-CTX Car3 Glut_2
COAa-PAA-MEA Barhl2 Glut_1
IT AON-TT-DP Glut_1
IT AON-TT-DP Glut_3
IT AON-TT-DP Glut_5
IT EP-CLA Glut_1
IT EP-CLA Glut_2
IT EP-CLA Glut_3
L2/3 IT ENT Glut_6
L2/3 IT PIR-ENTl Glut_1
L2/3 IT PIR-ENTl Glut_2
L2/3 IT PIR-ENTl Glut_3
L2/3 IT PIR-ENTl Glut_4
L2/3 IT PIR-ENTl Glut_6
L2/3 IT PIR-ENTl Glut_7
L6b EPd Glut_2
LA-BLA-BMA-PA Glut_2
MEA Slc17a7 Glut_2
K
100%
0%
% co-clustering in cluster
Cell type
L2/3 IT PIR-ENTl Glut_3
Ncam2+ PYR
L2/3 IT PIR-ENTl Glut_1
L2/3 IT ENT Glut_6
L2/3 IT PIR-ENTl Glut_6
IN_MGE
IT AON-TT-DP Glut_5
IT AON-TT-DP Glut_3
IT EP-CLA Glut_1
IN_CGE
L2/3 IT PIR-ENTl Glut_2
Abi3bp+ PYR
L2/3 IT PIR-ENTl Glut_7
IT AON-TT-DP Glut_1
OPC
Oligo
Rab3b+ SL
L2/3 IT PIR-ENTl Glut_4
Reln+ SL
IT EP-CLA Glut_3
LA-BLA-BMA-PA Glut_2
IT EP-CLA Glut_2
CLA-EpD-CTX Car3 Glut_2
CLA-EpD-CTX Car3 Glut_1
Micro
Astro
MEA Slc17a7 Glut_2
COAa-PAA-MEA Barhl2 Glut_1
Vglut2
L6b Epd Glut_2
16 13 18 4 10 3 2 19 12 11 8 5 15 7 17 6 14 1 0 9
Integrated clusters
